# Association of the Stroke Ready Community-Based Participatory Research Intervention With Incidence of Acute Stroke Thrombolysis in Flint, Michigan

**DOI:** 10.1001/jamanetworkopen.2023.21558

**Published:** 2023-07-03

**Authors:** Lesli E. Skolarus, Sarah Bailey, Casey L. Corches, Anne E. Sales, Chun Chieh Lin, Ran Bi, Mellanie V. Springer, Alina Oliver, Maria Cielito Robles, Tia Brooks, Michael Tupper, Michael Jaggi, Mohammed Al-Qasmi, Bruce A. Trevithick, Kimberly Barber, Aniel Majjhoo, Marc A. Zimmerman, William J. Meurer, Devin L. Brown, Lewis B. Morgenstern, James F. Burke

**Affiliations:** 1 Davee Department of Neurology, Stroke and Vascular Neurology, Northwestern University, Chicago, Illinois; 2 Department of Neurology, University of Michigan, Ann Arbor; 3 Bridges into the Future, Flint, Michigan; 4 Department of Family and Community Medicine, Sinclair School of Nursing, University of Missouri, Columbia; 5 VA Ann Arbor Healthcare System, Ann Arbor, Michigan; 6 Department of Neurology, Ohio State University, Columbus; 7 Bethlehem Temple Church, Flint, Michigan; 8 Department of Emergency Medicine, University of Michigan, Ann Arbor; 9 Department of Emergency Medicine, Hurley Medical Center, Flint, Michigan; 10 Genesee County Medical Authority, Flint, Michigan; 11 Department of Clinical & Academic Research, Genesys Regional Medical Center, Grand Blanc, Michigan; 12 Department of Neurology, McLaren Flint Hospital, Flint, Michigan; 13 School of Public Health, University of Michigan, Ann Arbor

## Abstract

**Importance:**

Acute stroke treatment rates in the US lag behind those in other high-income nations.

**Objective:**

To assess whether a hospital emergency department (ED) and community intervention was associated with an increased proportion of patients with stroke receiving thrombolysis.

**Design, Setting, and Participants:**

This nonrandomized controlled trial of the Stroke Ready intervention took place in Flint, Michigan, from October 2017 to March 2020. Participants included adults living in the community. Data analysis was completed from July 2022 to May 2023.

**Intervention:**

Stroke Ready combined implementation science and community-based participatory research approaches. Acute stroke care was optimized in a safety-net ED, and then a community-wide, theory-based health behavior intervention, including peer-led workshops, mailers, and social media, was conducted.

**Main Outcomes and Measures:**

The prespecified primary outcome was the proportion of patients hospitalized with ischemic stroke or transient ischemic attack from Flint who received thrombolysis before and after the intervention. The association between thrombolysis and the Stroke Ready combined intervention, including the ED and community components, was estimated using logistic regression models, clustering at the hospital level and adjusting for time and stroke type. In prespecified secondary analyses, the ED and community intervention were explored separately, adjusting for hospital, time, and stroke type.

**Results:**

In total, 5970 people received in-person stroke preparedness workshops, corresponding to 9.7% of the adult population in Flint. There were 3327 ischemic stroke and TIA visits (1848 women [55.6%]; 1747 Black individuals [52.5%]; mean [SD] age, 67.8 [14.5] years) among patients from Flint seen in the relevant EDs, including 2305 in the preintervention period from July 2010 to September 2017 and 1022 in the postintervention period from October 2017 to March 2020. The proportion of thrombolysis usage increased from 4% in 2010 to 14% in 2020. The combined Stroke Ready intervention was not associated with thrombolysis use (adjusted odds ratio [OR], 1.13; 95% CI, 0.74-1.70; *P* = .58). The ED component was associated with an increase in thrombolysis use (adjusted OR, 1.63; 95% CI, 1.04-2.56; *P* = .03), but the community component was not (adjusted OR, 0.99; 95% CI, 0.96-1.01; *P* = .30).

**Conclusions and Relevance:**

This nonrandomized controlled trial found that a multilevel ED and community stroke preparedness intervention was not associated with increased thrombolysis treatments. The ED intervention was associated with increased thrombolysis usage, suggesting that implementation strategies in partnership with safety-net hospitals may increase thrombolysis usage.

**Trial Registration:**

ClinicalTrials.gov Identifier: NCT036455900

## Key Points

**Question:**

Is optimizing acute stroke emergency department care coupled with a community stroke preparedness intervention associated with increased thrombolysis treatment rates in a predominantly Black community?

**Findings:**

In this nonrandomized controlled trial of the Stroke Ready intervention, which involved 5970 adults, optimizing emergency department care was associated with an increase in stroke thrombolysis treatment rates over time in the community.

**Meaning:**

These findings suggest that implementation science strategies in low-resource hospitals may be a good way to increase stroke thrombolysis treatments and promote health equity.

## Teaser

This nonrandomized controlled trial investigates whether a hospital emergency department and community intervention in Flint, Michigan, was associated with an increased proportion of patients with stroke receiving thrombolysis.

## Introduction

Administration of thrombolysis for acute ischemic stroke reduces poststroke disability, yet it is underused.^1^ Thrombolysis is time dependent, and administration requires a complex multidisciplinary care team often involving emergency medical services (EMS) professionals, nurses, emergency department (ED) physicians, and neurologists. Decreasing the time from stroke symptom onset to the presentation at the hospital (prehospital delay) and optimizing ED processes for acute stroke treatment could increase the number of patients with stroke eligible to receive thrombolysis and improve the effectiveness of acute stroke treatments.^2,3,4^

Acute stroke treatment is a model for the codependence of the community and the medical system. Patients with stroke must arrive at the ED soon after stroke symptom onset, and the ED must be optimized to treat them. Prehospital delay is predominantly due to patient-related factors, such as decision delay (ie, the time from symptom onset to the decision to seek medical attention) or the presence of stroke bystanders, given that patients with stroke may be unable to seek help.^5^ Although stroke is a common condition, the absolute lifetime risk of stroke for most individuals is low, so efforts to increase stroke preparedness, recognition of stroke warning signs, and the importance of calling EMS must be made community wide. Prehospital-focused interventions depend on receiving optimized acute stroke care upon hospital arrival. ED quality improvement initiatives have been promising,^6,7^ yet less is known about such initiatives in lower-resource settings.

Given the existence of stroke racial disparities, complexities of racial and income discrimination, and mistreatment within disinvested communities, it is crucial to partner with the community and local health system to design and test interventions. In this context, we present the results of the Stroke Ready project, an ED and community intervention to increase thrombolysis treatment by decreasing prehospital delay and optimizing ED acute stroke care developed and implemented through a community-based participatory research approach. We also hypothesized that Stroke Ready would increase EMS utilization and decrease the time from ED arrival to thrombolysis for stroke in Flint, Michigan.

## Methods

### Design

We conducted a nonrandomized controlled trial evaluation of the Stroke Ready program (eFigure in Supplement 1; original protocol in Supplement 2). The institutional review board from the University of Michigan granted a waiver of informed consent because the community stroke preparedness intervention presented no more than minimal risk of harm to participants and involved no procedures for which written consent is normally required outside the research context, in accordance with 45 CFR §46. We followed the Transparent Reporting of Evaluations With Nonrandomized Designs (TREND) reporting guideline for nonrandomized designs.

### Study Setting

Stroke Ready was conducted in Flint, Michigan, home to approximately 61 750 adults. The city’s population is predominantly Black people (54%), and more than 40% of the population lives below the poverty level.^8^ Flint has the highest poverty rate among US cities, with at least 65 000 residents, and it experienced a water crisis.^9,10^ Three hospitals in Genesee County care for Flint residents. All 3 hospitals participated in Get with the Guidelines–Stroke and achieved primary stroke center or comprehensive stroke center status prior to the start of the ED intervention. Similarly, acute stroke activations were managed locally without telemedicine stroke support during the study period. In 2010, Flint had one of the lowest acute stroke treatment rates of any community its size in the US.^11^

### Stroke Ready Program Overview

Stroke Ready was conducted through a long-standing community-based participatory research partnership established in 2010. The overarching goal of the Stroke Ready partnership is to improve the health and well-being of the Flint community. The partnership is co-led by a vascular neurologist (L.E.S.) and a community leader (S.B.). The community advisory board consisted of academic researchers, community members, representatives from community organizations, all Flint hospitals, emergency medical services, the county health department, and city government (see Additional Contributions at the end of the article) who partnered on all project phases.

The Stroke Ready program was a multilevel intervention that included an ED-based, implementation science–based intervention to optimize acute stroke care and a multimedia community intervention to increase stroke preparedness.^12^ The ED intervention was conducted in the safety-net hospital only because it was the hospital perceived by the community to care for the highest-risk patients with stroke in Flint. Although the intervention was focused on the ED, some of its aspects spilled into inpatient and other hospital care locations. We began with an assessment of the determinants of acute stroke care based on the Tailored Implementation for Chronic Diseases framework; with engaged ED partners, we selected implementation strategies to address the determinants.^13,14^ These strategies, implemented from October 2017 to May 2018, included external facilitation, audit and feedback, and development of a learning health collaborative with representatives across all stages of the stroke pathway and roles (eMethods in Supplement 1). Ultimately, this combination of implementation strategies led to the learning health collaborative implementing additional strategies, including additional quality monitoring, educational sessions, changing physical structure and equipment, and revising professional roles.^14,15^

From June 2018 to March 2020, we conducted a community-wide stroke preparedness intervention guided by the Theory of Planned Behavior, Social Cognitive Theory, and Behavioral Economics to reach adults in Flint.^16^ The community intervention consisted of peer-led individual and group education workshops with 5 standardized options ranging from 5 to 60 minutes delivered at community events, schools, community organization meetings, faith-based organizations, workplaces, senior centers, shelters, and beauty salons and barber shops. The workshops focused on subjective norms, outcome expectations, self-efficacy, precommitment, and nudges to call 911. Peer educators recorded observational demographics of participants. A total of 34 research team observations of peer educator–led workshops were conducted to assess intervention fidelity (eMethods in Supplement 1). None of the peer educators assessed required any additional training. In addition to the peer education, the community intervention consisted of (1) print materials, including mailers, brochures, and action plans (precommitment tool), and (2) digital, social, and broadcast media campaigns, including Stroke Ready website, music video, Facebook posts, Instagram posts, and public service announcements.^16^ The community intervention was rolled out sequentially across Flint quadrants over months and was intended to conclude in June 2020. However, given the restrictions imposed by the COVID-19 pandemic, the intervention ended in March 2020, shortening intervention and data collection time by 3 months.

### Data Sources, Study Population, and Outcomes

Multiple data sources and study populations were used to evaluate the results of Stroke Ready. The primary data source was the electronic medical record and administrative data from all Genesee County hospitals, accounting for 96.2% (3579 of 3718 treatments) of all thrombolysis treatments among Flint residents. Stroke included ischemic stroke (*International Classification of Diseases, Ninth Revision [ICD-9]* codes 433.x1, 434.x1, and 436; *International Classification of Diseases, Tenth Revision [ICD-10]* code I63*), transient ischemic attack (TIA) (*ICD-9* code 435; *ICD-10* code G45*), intracerebral hemorrhage (*ICD-9* code 431; *ICD-10* code I61*), and subarachnoid hemorrhage (*ICD-9* code 430; *ICD-10* code I60*). Patient demographics (including race and ethnicity), stroke type, insurance status, EMS arrival (10.2% missing), and acute stroke treatment were abstracted from the medical record. Because of the changing definition of stroke and TIA during the study period, thrombolysis outcomes were assessed among all patients with ischemic stroke or TIA.^17,18^ Addresses of patients with stroke and TIA were geocoded to identify Flint residents. The eligibility criteria for endovascular treatment evolved during the study period.^1^ Thus, the primary outcome was stroke thrombolysis (Medicare Severity Diagnosis-Related Group 61-63, *ICD-9 *procedure code 99.10, *ICD-10* procedure code 3E03317, *ICD-9* diagnosis code V45.88, or *ICD-10* diagnosis code Z92.82) among patients with ischemic stroke or TIA who resided in Flint and were admitted to 1 of the 3 hospitals. A secondary outcome included endovascular treatment (Medicare Severity Diagnosis-Related Group 22-24; *Current Procedural Terminology* codes 37184-6, 37201, 75896, and 61645; *ICD-9* procedure code 39.74; *ICD-10* procedure code 03CG3ZZ) or the combination of thrombolysis and endovascular treatment. Another secondary outcome was the proportion of patients with stroke (ie, ischemic stroke, TIA, intracerebral hemorrhage, or subarachnoid hemorrhage, because all have similar presentations) who arrived by EMS. In the safety-net hospital’s Get With the Guidelines database, we explored a secondary outcome of time from ED arrival to thrombolysis treatment among patients with ischemic stroke. The final data set was the Michigan State Inpatient Database from 2010 to 2020, which includes all acute care hospitalizations in Michigan within a given year. Demographics, stroke type, and thrombolysis by the city were ascertained.

### Sample Size

To maximize statistical power, the prespecified primary analysis parameterized the combined intervention, ED, and community components as a single variable. On the basis of estimates of Flint stroke admissions, with a preintervention Medicare treatment rate of 2.2%,^6,11,19^ we estimated we would have greater than 90% power to detect a doubling in the proportion of patients treated with thrombolysis considering a 2-sample binomial difference in proportions.

### Statistical Analysis

Descriptive statistics were used to describe the reach of the community intervention and the preintervention and postintervention population. The primary analysis was an interrupted time series design. The preintervention period was defined as 87 months before the start of the intervention (October 2017). Logistic regression was used to estimate the overall intervention association (indicator variable) in a model estimating receipt of thrombolysis (binary variable) accounting for stroke type (TIA vs ischemic stroke), time (month since start of intervention), and clustering at the hospital level (as a random effect). In a secondary analysis, the intervention period was separated into the ED and community intervention periods. We did not use this for our primary analysis because we suspected we would be underpowered to identify independent associations. We parameterized the ED intervention as turning on and sustaining over the entire period (binary variable representing periods before vs after the ED intervention) and the community intervention as turning on and changing over time as more people were reached with the sequential quadrant rollout (continuous variable representing months from the start of the community intervention period).^20^ We summarized model outcomes using average marginal effects. Sensitivity analyses assessed the robustness of our findings to different modeling assumptions: limiting the study population to only patients with ischemic stroke, expanding the study population to include all patients admitted to all Genesee county hospitals, accounting for patient factors associated with thrombolysis (ie, age, race [Black vs White and any other other race and ethnicity], and insurance status) and the outcome to include patients treated with thrombolysis and/or endovascular thrombectomy. Sensitivity analysis limited to Flint residents in the Michigan State Inpatient Database were also performed.

Additional secondary analyses explored how likely the association between Stroke Ready and outcomes were to be causal. Because the community intervention was delivered sequentially by Flint geographic quadrants, we explored whether increases in acute thrombolysis treatment rates paralleled the geographic pattern of intervention rollout by including an indicator of months from quadrant intervention. Finally, we conducted a logistic regression to examine thrombolysis trends in Flint vs other large Michigan metropolitan regions in which Black people comprise more than 25% of the population (Detroit, Saginaw, Muskegon, and Benton Harbor) to account for regional trends by using the Michigan State Inpatient Database.

For the secondary outcome of EMS utilization among all Flint patients with stroke, we performed a logistic regression model and accounted for known demographics associated with EMS utilization, including age, insurance status (insured vs not insured), and race (Black vs other race and ethnicity).^21^ For the secondary outcome of time from ED arrival to thrombolysis treatment among patients with ischemic stroke at the intervention ED, we explored the association of log-transformed time from ED arrival to thrombolysis treatment and the ED intervention adjusting for time, sex, age, and race (Black vs other race and ethnicity). All model covariates were considered statistically significant with a 2-sided *P* < .05. Statistical analyses were performed using SAS statistical software version 9.4 (SAS Institute) and Stata statistical software version 16 (StataCorp) from July 2022 to May 2023.

## Results

### Reach of the Community Intervention

In total, 5970 people received in-person, peer-led stroke preparedness education, corresponding to 9.7% of the adult population in Flint.^8^ These were conducted by 28 peer educators over the course of 234 in-person workshops. The number of workshop attendees was inversely correlated with the workshop duration (eResults in Supplement 1 including social media reach). The most common type of stroke preparedness workshop was the 5-minute 1-to-1 workshops that were delivered to 3550 people (59.5%). Mailers were sent to 44 136 residences, 347 posters were displayed in the community, and 790 radio plays were on an urban gospel station and an urban adult contemporary station.

### Characterization of the Population With Stroke in Flint Hospitals

There were 3327 patients from Flint with ischemic stroke and TIA (1848 women [55.6%]; 1747 Black individuals [52.5%]; mean [SD] age, 67.8 [14.5] years), including 2305 in the preintervention period and 1022 in the postintervention period (Table 1). There were no statistically significant differences in race, sex, or insurance status between periods. A higher ratio of ischemic stroke to TIA patients existed in the postintervention than preintervention period.

**Table 1.** Flint Patient Characteristics Between July 2010 and March 2020

Characteristic	Patients, No. (%)			*P* value
	Total (N = 3327)	Preintervention, July 2010-September 2017 (n = 2305)	Postintervention, October 2017-March 2020 (n = 1022)	
Age, mean (SD), y	67.8 (14.5)	68.0 (14.6)	67.4 (14.2)	.31
Sex				
Male	1479 (44.4)	1016 (44.1)	463 (45.3)	.51
Female	1848 (55.6)	1289 (56.0)	559 (54.7)	
Race				
Black	1747 (52.5)	1233 (53.5)	514 (50.3)	.09
Other race and ethnicity^a^	1580 (47.5)	1072 (46.5)	508 (49.7)	
Insured				
No	64 (1.9)	46 (2.0)	18 (1.7)	.65
Yes	3263 (98.1)	2259 (98.0)	1004 (98.2)	
Stroke type				
Ischemic	2457 (73.9)	1634 (70.9)	823 (80.5)	<.001
Transient ischemic attack	870 (26.2)	671 (29.1)	199 (19.5)	

^a^ Other includes White and any other race or ethnicity.

### Results of Primary and Secondary Analyses

**Figure 1.**
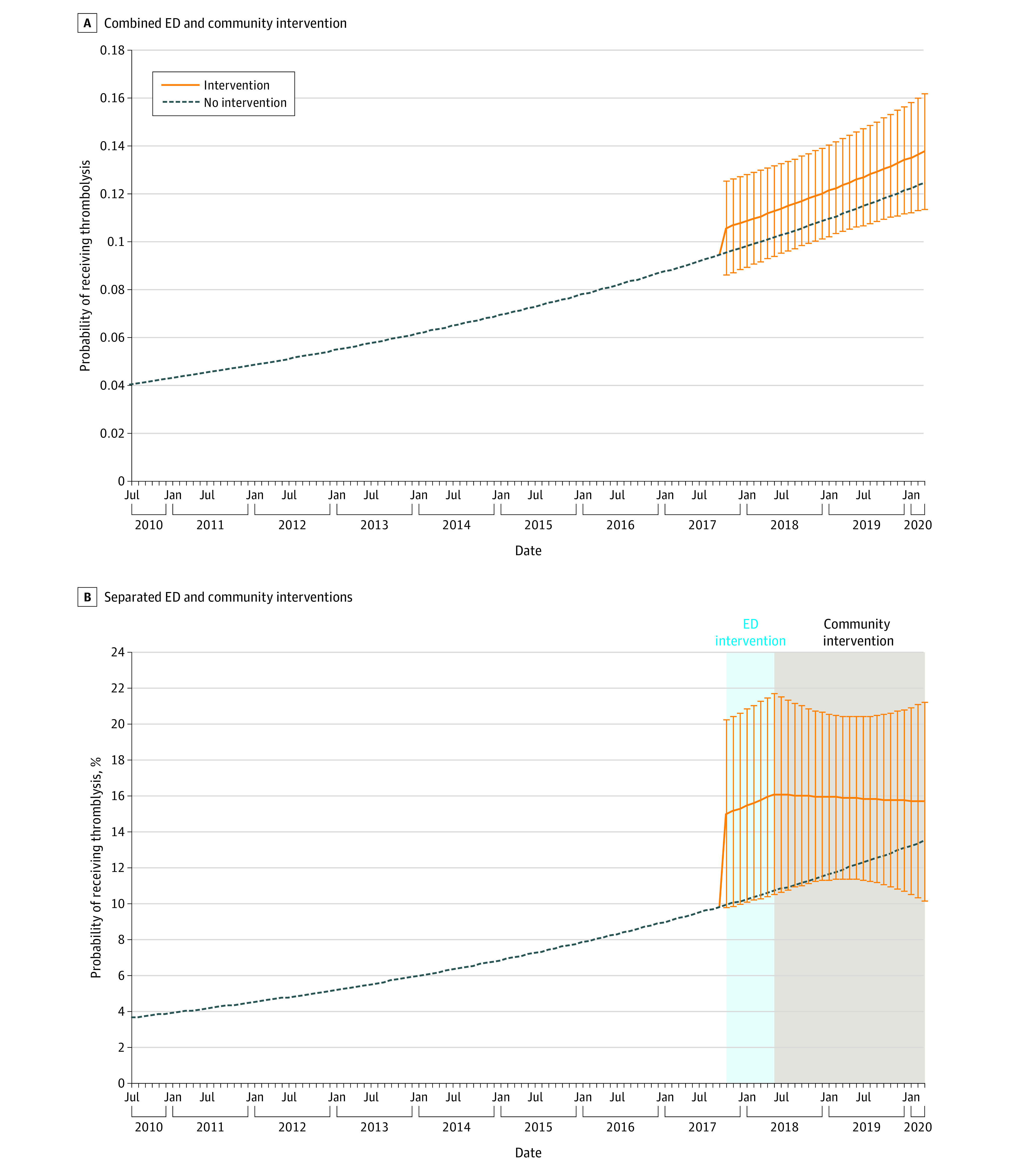
Proportion of Patients With Ischemic Stroke and Transient Ischemic Attack (TIA) Who Received Thrombolysis Over Time A, Proportion of patients with ischemic stroke and TIA who received thrombolysis over time compared with the combined emergency department (ED) and community Stroke Ready intervention, by month (clustered at hospital level, adjusted for time and stroke type). B, Proportion of patients with ischemic stroke and TIA who received thrombolysis over time compared with the separated Stroke Ready ED and community interventions by month (adjusted for time, stroke type, and hospital). In both panels error bars denote 95% CIs.

Thrombolysis increased from 4% in 2010 to 14% in 2020 among patients with ischemic stroke and TIA (adjusted OR per month, 1.01; 95%, CI, 1.00-1.02; *P* = .003) (Table 2 and Figure 1A). The combined ED and community intervention was not associated with thrombolysis (OR, 1.13; 95% CI, 0.74-1.70; *P* = .58). The associations differed by intervention component (Table 2). The ED intervention component alone was associated with an increased likelihood of thrombolysis (OR, 1.63; 95% CI, 1.04-2.56; *P* = .03) (Table 2 and Figure 1B), suggesting that the ED intervention was associated with an increase in thrombolysis rates to 15% of patients with ischemic stroke or TIA from the 10%, which would have been projected on the basis of temporal trends alone. The community intervention component alone was not associated with an increased likelihood in thrombolysis (OR, 0.99; 95% CI, 0.96-1.01; *P* = .30) (Table 2 and Figure 1B). There was no association between quadrant activity and receipt of thrombolysis (OR, 1.00; 95% CI, 0.97-1.03; *P* = .85) (eTable 1 in Supplement 1). Sensitivity analyses that accounted for patient demographics, limited the population to only patients with ischemic stroke, or expanded the study population to include all patients admitted to Flint hospitals or patients who received EVT and Flint patients in the Michigan State Inpatient Database showed similar results (eTables 1-5 in Supplement 1).

**Table 2.** Stroke Ready Intervention and Likelihood of Receiving Thrombolysis Among Patients in Flint, Michigan (N = 3327)

Model and variable	OR (95% CI)	*P* value
Model 1: combined ED and community intervention^a^		
Change in thrombolysis since January 2010, per mo	1.01 (1.00-1.02)	.003
Combined intervention (after vs before)	1.13 (0.74-1.70)	.58
Model 2: separate ED and community intervention^b^		
Change in thrombolysis since January 2010, per mo	1.01 (1.01-1.02)	<.001
ED intervention	1.63 (1.04-2.56)	.03
Change in thrombolysis after community intervention, per mo	0.99 (0.96-1.01)	.30

Abbreviations: ED, emergency department; OR, odds ratio.

^a^ Model 1 was adjusted for stroke type and clustering by hospital.

^b^ Model 2 was adjusted for stroke type and hospital.

**Figure 2.**
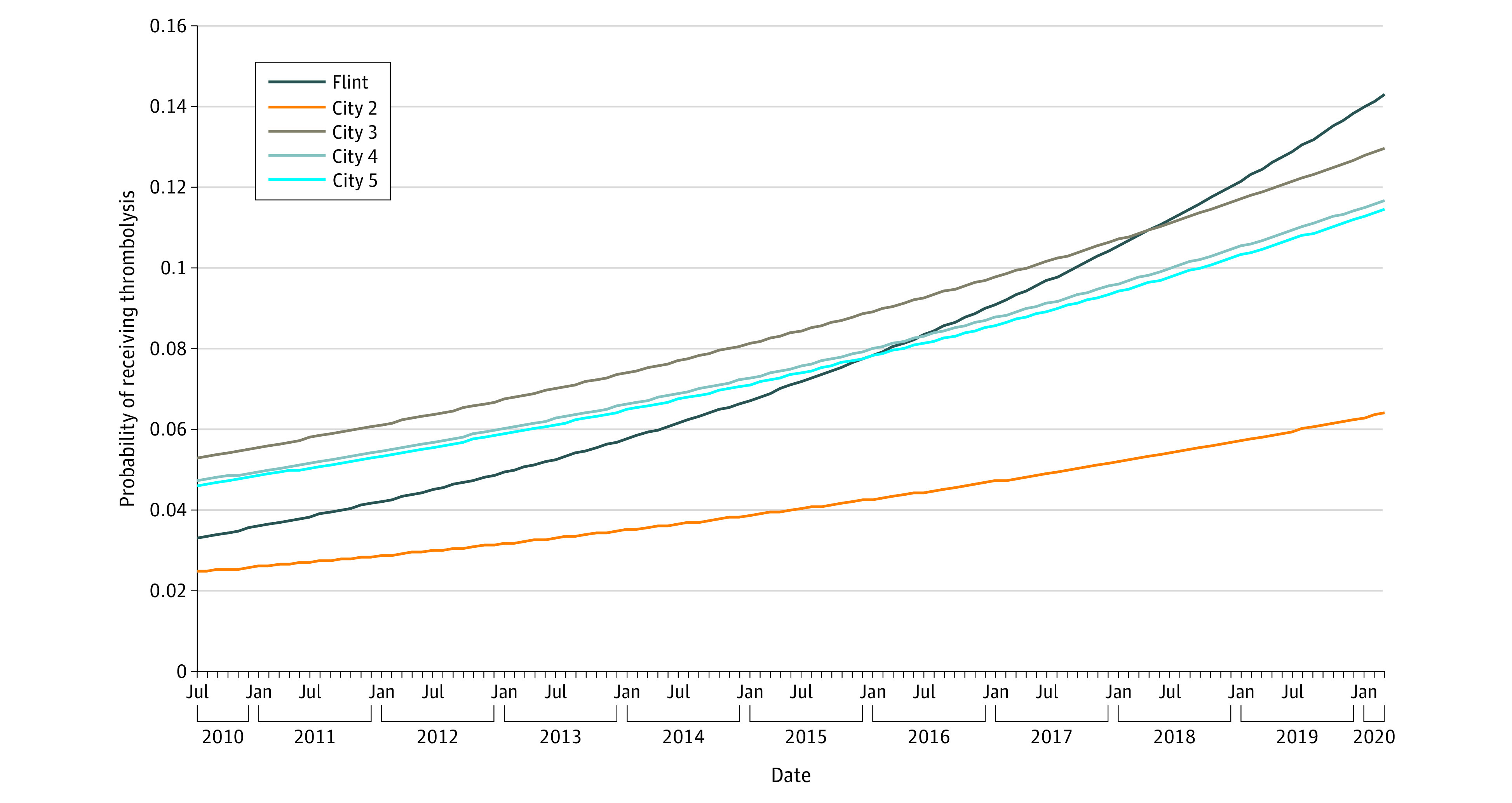
Proportion of Patients With Ischemic Stroke and Transient Ischemic Attack Who Received Thrombolysis by Michigan City by Month Data were adjusted for stroke type.

The proportion of Black patients with stroke among the Michigan comparator cities ranged from 23% to 85% (eTable 6 in Supplement 1). Thrombolysis increased over time across the 5 communities (adjusted OR per month, 1.01; 95% CI, 1.01-1.01; *P* < .001) (Figure 2) Thrombolysis rates increased faster in Flint than in the other communities (adjusted OR per month, 1.01; 95% CI, 1.00-1.01; *P* = .006) (Figure 2). In 2010, Flint had the second lowest community thrombolysis treatment rates and increased to the highest proportion of patients with stroke or TIA treated with thrombolysis in 2020.

### Changes in EMS Use Over Time in Flint and ED Arrival to Thrombolysis

EMS arrival rates for patients with stroke and TIA increased from 49% in 2010 to 58% in 2020 (adjusted OR, 1.00; 95% CI, 1.00-1.01; *P* = .03) (eTable 3 in Supplement 1). There was no association between the community intervention and EMS use (adjusted OR, 1.00; 95% CI, 0.99-1.02; *P* = .89) (eTable 7 in Supplement 1). The median (IQR) time from ED arrival to thrombolysis was 80 (59-98) minutes in the preintervention period and 58 (40-85) minutes in the postintervention period, a 22-minute decrease, although the percentage decrease was not significant (−23%; 95% CI, −47% to 1%; *P* = .05) (eTables 7 and 8 in Supplement 1).

## Discussion

Stroke Ready, a multilevel ED optimization and community stroke preparedness intervention, was not associated with increased use of thrombolysis. However, when analyzing the Stroke Ready intervention components separately, we found that the ED intervention was associated with increased thrombolysis, but the community intervention was not. In the last decade, Flint improved from one of the lowest-performing US communities for delivering stroke thrombolysis to a higher-performing community.

Stroke Ready did not increase thrombolysis more than would be expected by the time trend in Flint alone. The failure to detect an association may be related to trends in thrombolysis that were insufficiently accounted for in our study design. There has been a consistent worldwide increase in thrombolysis. In 2009, the US thrombolysis rate was approximately 4.6%, compared with approximately 11% in 2018, and varied across communities.^11,22^ In planning Stroke Ready in 2015, we powered the study for a relative 100% increase (ie, a doubling) in thrombolysis treatment rates or an absolute increase of 2% from the low baseline 2% treatment rate observed in Flint.^12^ The absolute observed change in thrombolysis rate over the study period was, in fact, 10%. As such, we were substantially underpowered to differentiate the effect of Stroke Ready from the robust observed time trend.

The Stroke Ready ED intervention was associated with a statistically significant increase in thrombolysis and decreased time from ED arrival to thrombolysis. Notably, this increase was found in hospitals that participated in Get With the Guidelines–Stroke and had achieved stroke center certification. Cluster-randomized ED thrombolysis implementation trials in the US, Australia, and the Netherlands have shown small, non–statistically significant increases in use of thrombolysis.^23,24,25^ These trials did not specifically select EDs with low thrombolysis usage, and in fact, 1 trial^25^ found no change in thrombolysis in EDs that were higher users of thrombolysis at baseline, suggesting that some EDs may be at their capacity. Selecting low-performing EDs leads to a larger opportunity to improve and maybe one approach to future interventions to increase thrombolysis.

The Stroke Ready community intervention was not associated with thrombolysis treatment rates. In addition to the power concerns, several points suggest a more cautious interpretation of the findings: (1) our study design precludes true independent evaluation of the hospital and community intervention (eg, the community intervention may have led to more thrombolysis-eligible patients with stroke presenting to the safety-net ED); (2) the community intervention and data collection were shortened by 3 months because of research restrictions necessitated by COVID-19^26,27^; and (3) thrombolysis in Flint increased faster than in comparator Michigan cities, suggesting that Stroke Ready might have had some benefit not directly accounted for by our analysis. However, our findings are consistent with recent community intervention studies that found no benefit on prehospital delay, EMS use, or thrombolysis,^28,29^ except in communities with low baseline stroke preparedness.^30^ Furthermore, community stroke preparedness interventions are resource intensive, and the long-term fidelity and sustainability are uncertain. Thus, moving forward more effective community stroke preparedness interventions are needed.

Stroke Ready highlights some of the strengths and challenges in performing complex, multilevel interventions in the context of a community-based participatory research partnership. Strengths of the community intervention include the depth of community engagement, including community ideation, intervention development guided by health behavior theory, pilot testing with Black churches, delivery of stroke preparedness intervention by peer educators to facilitate sustainability, and co-led results dissemination.^12,31^ Our community-engaged approach likely facilitated the substantial reach of Stroke Ready into the community (ie, 10% of the Flint adult population received an in-person intervention). Challenges include the scale needed to test community-level interventions. The ideal study design would be a cluster-randomized trial of matched communities across the US, yet this requires multiple, often at least 10, communities to attain statistical power. These trials also require substantial resources, including the engagement of community partners across communities and an evidence base to suggest that the trial resources are warranted.^32,33^

### Limitations

There are additional limitations of our study worth considering. First, this was a single community, and thus our results may not be generalizable to other cities. Second, data were not available on patient eligibility for thrombolytics, including stroke onset to ED arrival times. Data on whether Flint patients with stroke or their social networks were exposed to Stroke Ready were also unavailable. Third, stroke and TIA diagnosis and acute stroke outcomes were obtained via diagnosis codes from the medical record. Fourth, we do not have measures of EMS use appropriateness. Fifth, the secondary outcome of EMS use was limited by missing data slightly greater in the postintervention period. Missing EMS data are more likely among patients who do not arrive to the ED by EMS and, thus, should be biased toward the null.

## Conclusions

This nonrandomized controlled trial found that the combined Stroke Ready intervention was not associated with increased thrombolysis rates, but the results suggest that the Stroke Ready ED intervention is promising. From a community perspective, Flint’s substantial increase in thrombolysis rates over the last decade should be celebrated. Future studies should test the efficacy of the Stroke Ready ED intervention in other communities and using other study designs.

## Notes

Supplement 1. **eFigure.** Stroke Ready Study Flow
eMethods.

eResults.
**eTable 1.** Predicting the Likelihood of Receiving Thrombolysis Among Flint Patients (N=3327) **eTable 2.** Predicting the Likelihood of Receiving Thrombolysis Among Flint Patients With Ischemic Stroke (N=2457) **eTable 3.** Predicting the Likelihood of Receiving Thrombolysis Among Patients Admitted to All Flint Hospitals (N=5474) **eTable 4.** Predicting the Likelihood of Receiving Thrombolysis or Endovascular Therapy Among Flint Patients (N=3327) **eTable 5.** Predicting the Likelihood of Receiving Thrombolysis Among Flint Patients Using the Michigan State Inpatient Database (N=3755) **eTable 6.** Factors Associated With Arriving by EMS **eTable 7.** Patient Characteristics From 5 Cities in Michigan Between July 2010 and March 2020 **eTable 8.** Stroke Ready ED Intervention and Time From ED Arrival to Thrombolysis (n = 183)
eReferences

Supplement 2.
Trial Protocol

Supplement 3.
Data Sharing Statement
