# Supplementary material for: Association of the Stroke Ready Community-Based Participatory Research Intervention With Incidence of Acute Stroke Thrombolysis in Flint, Michigan
Source: JAMA Netw Open. 2023 Jul 3;6(7):e2321558. doi: 10.1001/jamanetworkopen.2023.21558 (PMC10318478; doi:10.1001/jamanetworkopen.2023.21558)
Supplement: Supplement 1. — eFigure. Stroke Ready Study Flow eMethods. eResults. eTable 1. Predicting the Likelihood of Receiving Thrombolysis Among Flint Patients (N=3327) eTable 2. Predicting the Likelihood of Receiving Thrombolysis Among Flint Patients With Ischemic Stroke (N=2457) eTable 3. Predicting the Likelihood of Receiving Thrombolysis Among Patients Admitted to All Flint Hospitals (N=5474) eTable 4. Predicting the Likelihood of Receiving Thrombolysis or Endovascular Therapy Among Flint Patients (N=3327) eTable 5. Predicting the Likelihood of Receiving Thrombolysis Among Flint Patients Using the Michigan State Inpatient Database (N=3755) eTable 6. Factors Associated With Arriving by EMS eTable 7. Patient Characteristics From 5 Cities in Michigan Between July 2010 and March 2020 eTable 8. Stroke Ready ED Intervention and Time From ED Arrival to Thrombolysis (n = 183) eReferences [file jamanetwopen-e2321558-s001.pdf]

Supplemental Online Content

Skolarus LE, Bailey S, Corches CL, et al. Association of the Stroke Ready community-based participatory research intervention with incidence of acute stroke thrombolysis in Flint, Michigan. *JAMA Netw Open*. 2023;6(7):e2321558. doi:10.1001/jamanetworkopen.2023.21558

eFigure. Stroke Ready Study Flow

eMethods.

eResults.

eTable 1. Predicting the Likelihood of Receiving Thrombolysis Among Flint Patients

(N=3327)

eTable 2. Predicting the Likelihood of Receiving Thrombolysis Among Flint Patients With Ischemic Stroke (N=2457)

eTable 3. Predicting the Likelihood of Receiving Thrombolysis Among Patients Admitted to All Flint Hospitals (N=5474)

eTable 4. Predicting the Likelihood of Receiving Thrombolysis or Endovascular Therapy Among Flint Patients (N=3327)

eTable 5. Predicting the Likelihood of Receiving Thrombolysis Among Flint Patients Using the Michigan State Inpatient Database (N=3755)

eTable 6. Factors Associated With Arriving by EMS

eTable 7. Patient Characteristics From 5 Cities in Michigan Between July 2010 and March 2020

eTable 8. Stroke Ready ED Intervention and Time From ED Arrival to Thrombolysis (n = 183)

eReferences

This supplemental material has been provided by the authors to give readers additional information about their work.

© 2023 Skolarus LE et al. *JAMA Network Open.*

(eFigure) Stroke Ready Study Flow

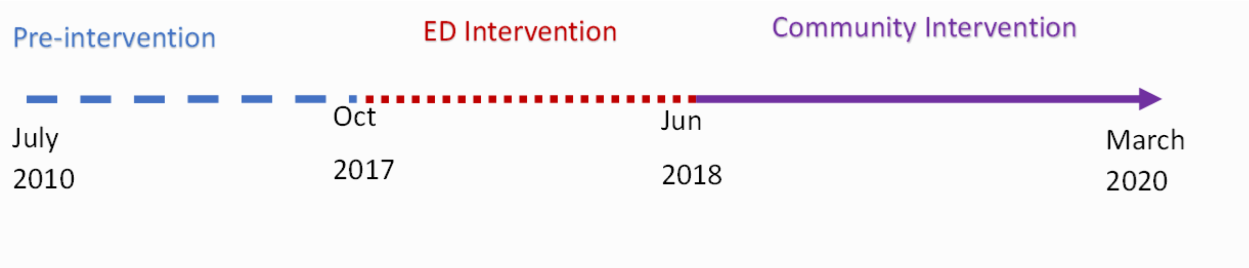

eMethods

The academic team performed external facilitation1. It included participatory design of feedback reports, audit and feedback report creation, troubleshooting, sharing expertise and clinical resources (i.e., protocols, etc.). Monthly audit and feedback reports began with stroke education, including the acute stroke treatment process and outcome metrics. These were supplemented with near real-time emails to the treating medical team and patient follow-up discussions at learning health collaborative meetings and team huddles to heighten awareness of acute stroke outcomes. The learning health collaborative met monthly to review the feedback report of each acute case and engage in collaborative deliberation of innovative strategies to overcome existing barriers.[1]

Fidelity

The research team assessed individual-level intervention fidelity regularly to ensure that the intervention components were being implemented as designed, and that there was consistency in delivery across peer educators. Fidelity was assessed using an observation form to measure adherence to intervention length, content, methods, activities, facilitation quality, and participant responsiveness. Notes were taken during each observation to record any environmental aspects that may have influenced intervention implementation or study outcomes. Peer educators were also provided immediate feedback about their performance including areas of strength and suggestions for improvement.

eResults

The most common in-person stroke preparedness education was the one-to-one 5-minute sessions conducted with 3,550 people (59.5%); while 200 (3.4%) received the 60-minute group session. A total of 36% of workshops were attended by primarily Black participants, 18% by primarily White participants, and 46% by both Black and White participants. Most workshops included both men and women (71%) and participants who were middle-aged (41-64 years, 58%).

We created 160 Facebook posts, resulting in 184 followers and engagement (reactions, comments, shares) with 3,082 people, and Stroke Ready content reached 10,848 people living in Flint. We created 75 Instagram posts, resulting in 122 followers and reaching 1,012 people. Finally, our website had 1,100 visitors and included our 3 music videos (Think FAST, American Sign Language,[2] stroke preparedness during the COVID-19 pandemic).[3]

eTable 1. Predicting the likelihood of receiving thrombolysis among Flint patients (N=3327)

**Community Quadrants***				**Accounting for patient factors***		
	OR (95%CI)	p-value			OR (95%CI)	p-value
Change in thrombolysis since January 2010, per month	1.01 (1.005-1.02)	<0.01		Change in thrombolysis since January 2010, per month	1.01 (1.006-1.02)	<0.01
ED Intervention	1.56 (0.996-2.44)	0.052		ED Intervention	1.64 (1.04-2.58)	0.03
Months from Quadrant Intervention	0.997 (0.97-1.03)	0.85		Change in thrombolysis after community intervention per month	0.98 (0.96-1.008)	0.18
				Patient Factors		
				Insured (vs. not-insured)	1.14 (0.47-2.75)	0.78
				age	0.99 (0.98-0.997)	0.01
				Black (vs. non-Black)	0.62 (0.47-0.80)	<0.01

*adjusted for stroke type, hospital; p value was 2-sided.

eTable 2. Predicting the likelihood of receiving thrombolysis among Flint patients with ischemic stroke (N=2457)

**Combined ED and community intervention***				**Separate ED and community intervention***		
	OR (95%CI)	p-value			OR (95%CI)	p-value
Change in thrombolysis since January 2010, per month	1.01 (1.003-1.02)	<0.01		Change in thrombolysis since January 2010, per month	1.01 (1.006-1.02)	<0.01
Combined Intervention	1.21 (0.79-1.85)	0.38		ED Intervention	1.58 (0.999-2.5)	0.05
				Change in thrombolysis after community intervention, per month	0.99 (0.96-1.02)	0.48

*adjusted for stroke type, hospital; p value was 2-sided.

eTable 3. Predicting the likelihood of receiving thrombolysis among patients admitted to all Flint Hospitals (N=5474)

**Separate ED and community intervention***						
	OR (95%CI)	p-value				
Change in thrombolysis since January 2010, per month	1.009 (1.004-1.014)	<0.01				
ED Intervention	1.37 (0.962-1.952)	0.08				

© 2023 Skolarus LE et al. *JAMA Network Open.*

Flint*Change in thrombolysis after community intervention, per month	1 (0.978-1.022)	0.99				

*adjusted for stroke type, hospital; p value was 2-sided.

eTable 4. Predicting the likelihood of receiving thrombolysis or endovascular therapy among Flint patients (N=3327)						
**Combined ED and community intervention***				**Separate ED and community intervention***		
	OR (95%CI)	p-value			OR (95%CI)	p-value
Change in thrombolysis since January 2010, per month	1.02 (1.008-1.02)	<0.01		Change in thrombolysis since January 2010, per month	1.02 (1.01-1.02)	<0.01
Combined Intervention	0.88 (0.6-1.3)	0.53		ED Intervention	1.4 (0.91-2.15)	0.12
				Change in thrombolysis after community intervention, per month	0.974 (0.95-0.998)	0.03

*adjusted for stroke type, hospital; p value was 2-sided.

eTable 5. Predicting the likelihood of receiving thrombolysis among Flint patients using the Michigan State Inpatient Database (N=3755)						
**Combined ED and community intervention***				**Separate ED and community intervention***		
	OR (95%CI)	p-value			OR (95%CI)	p-value
Change in thrombolysis since January 2010, per month	1.01 (1.005-1.02)	<0.01		Change in thrombolysis since January 2010, per month	1.01 (1.008-1.02)	<0.01
Combined Intervention	1.06 (0.72-1.58)	0.76		ED Intervention	1.55 (0.977-2.37)	0.06
				Change in thrombolysis after community intervention, per month	0.985 (0.96-1.01)	0.25

*adjusted for stroke type, hospital; p value was 2-sided.

© 2023 Skolarus LE et al. *JAMA Network Open.*

eTable 6. Factors associated with arriving by EMS

	OR (95%CI)	p value
Change in thrombolysis since January 2010, per month	1.003 (1-1.006)	0.03
Change in thrombolysis after community intervention, per month	1.001 (0.985-1.017)	0.89
*also adjusted for hospitals, stroke type, patient insurance status, age and race		

eTable 7. Patient characteristics from 5 cities in Michigan between July 2010 and March 2020

MI residents in 5 cities	total	Flint	City 2	City 3	City 4	City 5	p-value
	N=31663	4509 (14.24)	886 (2.80)	3857 (12.18)	2850 (9.00)	19561 (61.78)	
	Mean (SD)	Mean (SD)	Mean (SD)	Mean (SD)	Mean (SD)	Mean (SD)	
Age	66.50 (14.6)	67.23 (14.5)	67.00 (13.8)	69.48 (14.7)	70.57 (14.6)	65.13 (14.4)	<.01
	N (%)	N (%)	N (%)	N (%)	N (%)	N (%)	
Stroke Type							<.01
Ischemic	23631 (74.6)	3285 (72.9)	722 (81.5)	2553 (66.2)	2291 (80.4)	14780 (75.6)	
TIA	8032 (25.4)	1224 (27.2)	164 (18.5)	1304 (33.8)	559 (19.6)	4781 (24.4)	
Gender							0.10
Men	14090 (44.5)	2004 (44.4)	439 (49.6)	1739 (45.1)	1291 (45.3)	8617 (44.1)	
Women	17570 (55.5)	2504 (55.5)	447 (50.5)	2118 (54.9)	1559 (54.7)	10942 (55.9)	
Missing	3 (0.01)	1 (0.02)	0 (0.00)	0 (0.00)	0 (0.00)	2 (0.01)	
Race							<.01
Non-Black	9336 (29.5)	2175 (48.24)	333 (37.58)	2565 (66.5)	2179 (76.5)	2084 (10.7)	
Black	21438 (67.7)	2270 (50.34)	547 (61.74)	1258 (32.66)	660 (23.2)	16703 (85.4)	
Missing	889 (2.8)	64 (1.42)	6 (0.7)	34 (0.9)	11 (0.4)	774 (4.0)	
Insured							<.01
No	770 (2.4)	95 (2.1)	22 (2.5)	74 (1.9)	30 (1.1)	549 (2.8)	
Yes	30893 (97.6)	4414 (97.9)	864 (97.5)	3783 (98.1)	2820 (99.0)	19012 (97.2)	

© 2023 Skolarus LE et al. *JAMA Network Open.*

eTable 8. Stroke Ready ED Intervention and Time From ED Arrival to Thrombolysis (n = 183)[a,b]

	P.E. (95% CI)	*P* value
Intercept	4.71 (4.19 to 5.22)	<.001
Change in thrombolysis since January 2010, per mo	0.00 (−0.01 to 0.00)	.30
ED intervention	−0.23 (−0.47 to 0.01)	.054

Abbreviation: ED, emergency department.
[a]Also adjusted patient sex, age and race (Black vs non-Black).
[b]Outcome is the log of time from ED to thrombolysis.

eReferences:

1. Springer MV, Sales AE, Islam N, McBride AC, Landis-Lewis Z, Tupper M, Corches CL, Robles MC, Skolarus LE. A step toward understanding the mechanism of action of audit and feedback: a qualitative study of implementation strategies. *Implementation Science*. 2021;16:1-13.
2. Springer MV, Corches CL, McCracklin B, Scott M, Robles MC, Skolarus LE. Expanding Stroke Preparedness to Vulnerable Populations: A Music Video for the Deaf Community. *Journal of the American Heart Association*. 2020;9:e018352. doi: doi:10.1161/JAHA.120.018352
3. Robles MC, Corches CL, Bradford M, Rice TS, Sukul D, Springer MV, Bailey S, Oliver A, Skolarus LE. Understanding and informing community emergency cardiovascular disease preparedness during the COVID-19 pandemic: stroke ready. *Journal of Stroke and Cerebrovascular Diseases*. 2021;30:105479.

© 2023 Skolarus LE et al. *JAMA Network Open.*
