# Supplementary material for: Association of the Stroke Ready Community-Based Participatory Research Intervention With Incidence of Acute Stroke Thrombolysis in Flint, Michigan
Source: JAMA Netw Open. 2023 Jul 3;6(7):e2321558. doi: 10.1001/jamanetworkopen.2023.21558 (PMC10318478; doi:10.1001/jamanetworkopen.2023.21558)
Supplement: Supplement 2. — Trial Protocol [file jamanetwopen-e2321558-s002.pdf]

Stroke Ready: A Community Based Participatory Trial to Increase Stroke Treatment

Stroke Ready is a health behavior theory-based, multi-level intervention, designed to increase acute stroke treatment in Flint. The aims of the grant are:

Specific Aim 1: To adapt and expand our CBPR-developed, theory-based, Stroke Ready pilot community intervention and implement a hospital-based intervention to optimize acute stroke care in an urban safety-net, hospital.

Specific Aim 2: To increase acute stroke treatment rates in Flint, Michigan through a two-pronged approach of hospital and community level interventions.

Specific Aim 3: To inform future CBPR acute stroke treatment interventions by exploring both the relative importance of community and hospital interventions and the efficacy of the intervention on processes mediating the outcome.

Our hospital-level intervention aims to optimize acute stroke hospital care in a safety net ED. This has been approved under HUM00112536. Meanwhile, the community intervention seeks to increase acute stroke treatment (by decreasing pre-hospital delay). Ultimately, by exploring the effects of the community and hospital interventions individually and together, Stroke Ready can serve as a model for other at risk communities to increase acute stroke treatment. We will also estimate the cost effectiveness which is vital as hospitals and communities determine how to use their resources and meet the requirement of stroke centers to provide community education. This application is for the community-level intervention.

Background

Post-stroke disability is common, costly and projected to increase. Most of the more than 7 million stroke survivors in the US have disability. Acute stroke treatments, which include intravenous tissue plasminogen activator (tPA) and intra-arterial treatment, substantially reduce post-stroke disability but are administered to less than 5% of stroke patients. These treatments are particularly underutilized in Flint, Michigan, where the rate of acute stroke treatment is half the national rate. In fact, Flint has the lowest treatment rate of any region of its size in the entire US, which only exacerbates the existing health disparities in this predominantly African-American community. The low treatment rates of the Flint community are illustrative of racial disparities in stroke — African Americans have a higher incidence of stroke, receive acute stroke treatments less often and experience greater post-stroke disability than non-Hispanic whites. These inequities can be at least partially addressed with interventions to increase acute stroke treatment rates; but practical, cost efficient and sustainable interventions are lacking.

Acute stroke treatments are administered in the Emergency Department and are both rigidly time limited and highly time sensitive. Earlier treatment means a greater chance of stroke recovery ("Time is brain"). One of the main reasons for acute stroke treatment underutilization is pre-hospital delay — patients arrive to the hospital too late to receive the treatment. One strategy to reduce pre-hospital delay is to focus on stroke preparedness (ability to recognize acute stroke symptoms and call 911 immediately) through community behavioral interventions. Over the past 7 years, the research teams composed of researchers from the University of Michigan and community partners from Flint, increased stroke preparedness in Flint through our Stroke Ready pilot intervention (HUM00098718). In addition to pre-hospital delays, hospital delays also contribute to Flint's low acute stroke treatment rates. When stroke patients arrive to the hospital, a multistep process occurs to determine whether the patient is eligible for acute stroke treatments and to rapidly administer the treatment. Hospitals are known to vary widely in their abilities to execute these complex treatment pathways and thus, optimal interventions to improve treatment rates should target both the community and the hospital.

Flint, the birthplace of General Motors, was once a thriving industrial city. Like many cities in the industrial Midwest, the collapse of the automobile industry has exacerbated the economic struggles of the city.[1] The majority of the population is African American (60%) and over 40% live below the poverty level. Flint is experiencing a water crisis due to high lead levels in the drinking water. It is vital to increase acute stroke treatments in Flint because: 1) Genesee County, where Flint is the largest city, has one of the highest age-adjusted stroke hospitalization rates in Michigan[2]; 2) Flint has the lowest acute stroke treatment rate of any community of its size in the US[3]. Nationally, a mean of 4.2% stroke patients receive acute stroke treatment, but, in Flint the mean treatment rate is 2.2% and the very best regions within Flint only perform at the national average; and 3) Hurley Medical Center, the site of the implementation strategies to optimize ED acute stroke care, is well below the national average in acute stroke treatment quality measures.[4] This study will inform future acute stroke interventions in underserved, predominately African American communities and safety-net hospitals. It will assist in determining the best use of often limited resources to increase stroke preparedness whether to community preparedness or hospital implementation.

Significance

With nearly 800,000 strokes in the US annually, stroke is a leading cause of disability.[5] The number of US stroke survivors is projected to increase from 7 to 10 million by 2030 given the aging baby boomer generation and declining stroke mortality.[5-8] Because most people survive their stroke, disability is the greatest challenge facing survivors and their families. About two-thirds of stroke survivors are left with disability.[9, 10] Acute stroke treatments reduce disability but are underutilized particularly in African Americans. Acute stroke treatments reduce the relative risk of post-stroke disability by over 30%.[11-14] The two main phases of stroke care are: 1) pre-hospital— time from when the patient is in the community and a stroke occurs to their arrival to the hospital; 2) hospital— time from stroke patient's arrival to the hospital to receiving acute stroke treatment in the ED. Both of these phases are fraught with delay. We conducted a systematic review of interventions to increase acute stroke treatment.[15] Most interventions are resource intensive, of uncertain cost effectiveness and difficult to justify in underserved communities. The major limitation of applying acute stroke interventions is the inclusion of community and hospital components resulting in large resource utilization.[16-25] Further, most stroke preparedness interventions have not been health theory based which may account for the decreased effectiveness among African Americans.[25-27] Stroke Ready is a practical and pragmatic approach to increasing acute stroke treatments, based on health behavior theory, that can be implemented in underserved minority communities. The result will be a rigorous, expertly crafted and conducted study that has the potential to provide an intervention that will reduce the disability and expense from stroke. This study will create stroke preparedness public health materials that can be used in wide-spread intervention efforts to reduce stroke risk.

Study Design and Overview

This application consists of two parts, a health education component which the research team believes is exempt; and obtaining hospital data which will contain PHI (subject addresses) and is non-exempt, regulated.

The Stroke Ready project is a quasi-experimental, health behavior theory based, hospital and community intervention designed to increase acute stroke treatments. The design of the community intervention will be sequentially delivered to each Flint quadrant, i.e. four clusters defined by the community's geographic boundaries, until all the quadrants have crossed over from control to intervention eventually reaching the entire community (Table 1). This strategy will allow the research team to observe the effects of the community intervention separately from the hospital intervention (HUM112536) to assess the impact of each intervention level. The community intervention consists of peer-led workshops, mailers, print, radio and internet media. (Details on study components are in the *Intervention Components* section of this protocol.)

Quad	Time 0	Time 1	Time 2	Time 3	Time 4
**1**	Pre-Intervention	**Workshop/Mailer Media: Radio/TV/Internet Poster/Brochure**	**Media: Radio/TV/Internet Poster/Brochure**	**Media: Radio/TV/Internet Poster/Brochure**	**Media: Radio/TV/Internet Poster/Brochure**
**2**	Pre-Intervention	Media: Radio/TV/Internet	**Workshop/Mailer Media: Radio/TV/Internet Poster/Brochure**	**Media: Radio/TV/Internet Poster/Brochure**	**Media: Radio/TV/Internet Poster/Brochure**
**3**	Pre-Intervention	Media: Radio/TV/Internet	Media: Radio/TV/Internet	**Workshop/Mailer Media: Radio/TV/Internet Poster/Brochure**	**Media: Radio/TV/Internet Poster/Brochure**
**4**	Pre-Intervention	Media: Radio/TV/Internet	Media: Radio/TV/Internet	Media: Radio/TV/Internet	**Workshop/Mailer Media: Radio/TV/Internet Poster/Brochure**

Table 1: Stroke Ready Community Component Roll Out

Inclusion/ Exclusion criteria (Stroke Ready workshops)

Workshops will be open to the public. While our primary outcome will observe Flint-based stroke treatment rates, stroke preparedness is a global public health message applicable to most people; therefore, participants will not be excluded if they live outside the city limits. Similarly, the workshops are designed for English-speaking adults; however, non-English speaking people will not be excluded. The Stroke Ready workshop should not be harmful to children as it is adapted from material delivered to adults and youth in the pilot study (HUM00098718). Thus, if adults choose to bring their children, they will be accommodated in the workshops.

Stroke Ready recruitment (Stroke Ready Workshops)

The research team and Stroke Ready peer-leaders will work together to recruit organizations and participants for the Stroke Ready workshops through recruitment announcements, flyers, internet platforms, and word-of-mouth. Since Flint is naturally divided into 4 quadrants the study team will select each quadrant for implementation and then for about the next 6 months they will focus their workshop recruitment efforts and material dissemination on the selected quadrant of the city. However, given the commitment to CBPR the quadrant implementation is preferred but we would not forego a workshop in a quadrant that we are not focusing on at the time if requested. During this time the research team and community partners, including peer leaders, will work together to implement the Stroke Ready workshops and distribute educational posters and materials. At the end of about six months, the materials will remain in the quadrant and efforts will shift to the next quadrant for recruitment and workshop delivery.

To overcome challenges of recruiting racial minorities, low-income individuals, and other disadvantaged populations due to lack of transportation, childcare, inconvenient hours, etc., the research team designed the workshops such that participants can participate at their convenience. The workshops are voluntary and will be delivered by peer-leaders at locations during times that are convenient for the community (e.g. after church services, during lunch breaks). Additional factors that facilitate program recruitment and participation are: 1) the workshops, as well as all Stroke Ready materials and activities, are free and have no more than minimal risk; 2) recruitment materials and intervention components were created by the community for the community; 3) the research team has gained support of local leadership and community members with strong ties in the Flint community; 4) children will be allowed to attend (HUM00098718).

Given the nature of the other community intervention components (i.e. educational posters for which recruitment is not applicable), the Stroke Ready workshop is the only component for which the research team and community partners will actively recruit, i.e. advertising its availability to the community. With regard to the music video, educational print materials, internet platforms, and mailers, there is no need to actively recruit as these interventions will be distributed and/ or implemented among the community as the others parts of the public health stroke preparedness message. With regard to workshop retention and compensation: Workshop participants will not be compensated for attending as this is a free, educational workshop on stroke preparedness. Depending on the location, participants may receive small tokens such as Stroke Ready pens of food. The workshop participant is free to leave the workshop at any time. The research team will oversee the activities related to workshop participant recruitment to track the campaign's reach and resources, and workshop delivery processes to assess workshop fidelity.

Stroke Ready Peer Leader

To build community capacity as well as to increase the uptake and relevance of the workshops in the community, peer leaders will be hired from the Flint community as much as possible for our community based intervention. We will recruit about 8-16 peer leaders to start but recognize there may be turnover during the intervention or high demand for additional workshops; therefore, the research team may need to recruit additional peer leaders during the workshop delivery phase. We will pay about $15 per hour for recruitment and delivering the workshops that include the minimum required attendees. We anticipate that some the peer leaders may be UM-Ann Arbor or UM-Flint students who prefer class credit to payment. If this were the case, we would work with the institution to facilitate credit attainment. Peer leaders will be cross-trained so that if one is unable to show up on the day of the workshop another peer leader will be able to facilitate the group. Peer leaders will be strongly encouraged workshops every week for the duration of the project.

The peer-leaders are not participants, but facilitators of the workshop. They will facilitate the workshop discussion and activities through use of a peer leader workshop facilitation guide and pre-recorded script. The research team will confirm peer leader workshop facilitation readiness by assessing Stroke Ready knowledge and Stroke Ready practice skill-set during the peer leader training session required by each peer leader before facilitating Stroke Ready workshops. Peer leaders will be evaluated on the materials to determine whether they have adequate knowledge to administer the workshop as well as the skills needed to facilitate the discussion and activities. The research team will train peer leaders in public presentation strategies, stroke educational materials, and other practices to build and strengthen a skillset to facilitate an open environment for learning and discussion. The research team will be available during business hours and as much as possible outside of business hours for any questions or concerns.

Stroke Ready Peer Leader Training

The Stroke Ready research team will provide workshop facilitation training to Stroke Ready peer leaders. The primarily face-to-face training will take approximately 8-10 hours (1-2 training sessions) and will be conducted in Flint, Michigan at a location and time agreed upon the research team and peer leaders. Subsequently, peer leaders will attain the Stroke Ready Peer Leader training completion certificate before the start of facilitating workshops in the community.

Peer leaders will be paid for completing the training. Upon training completion each peer leader will receive a trainer's guide, which provides a step-by-step teaching guide and peer leader script for the workshops, a tPA

demonstration kit, and the instructions for how to set up and use all workshop materials. All materials have been specifically tailored to the Flint community. At any point during the delivery of the workshops phase, the research team may determine that the peer leader needs additional training to fulfil the minimum requirements for the role. When additional training is needed, the research team will provide tailored training exercises and materials to the peer leader. The research team may not pay the peer leader for re-training. The research team may ask that the peer leader not facilitate additional workshops until they have completed the necessary retraining. Training and retraining activities will be tracked by the research team.

Intervention Components

The community intervention components focus on public health education emphasizing the significance of stroke preparedness. Primary Stroke Ready components will consist of peer facilitated educational workshops, a music video adapted and expanded from the pilot (HUM98718), a mass media campaign including print materials (e.g. posters, brochure), health promotion mailers, radio/TV public health service announcements, and the Stroke Ready website and Facebook page (Table 2). The community intervention components are purely public health education piloted in (HUM00098718) which includes stroke knowledge and stroke preparedness education.

We have submitted scripts/text for the stroke preparedness materials. These items are being edited/produced currently. We plan to submit to the IRBMED copies (or the equivalent) of the final materials when available. These will be complete with graphics, music, etc. We reviewed the content, graphics, and music with the community to assure appropriateness of content and cultural sensitivity. No participant is forced to watch or read any of the materials. They are provided to the community for them to use as they wish.

Table 2. Overview of Stroke Ready Components

Intervention components	Summary of intervention	Behavioral Construct	Focus Population	Delivery
**Peer-led Workshop and workbook**	Discussion and interactive stations	self-efficacy, attitude, social norms, knowledge	Churches, organizations, businesses	Peer leaders
**Music Video**	Educational music video of stroke preparedness	self-efficacy, attitude, social norms, knowledge	Churches, organizations, businesses, events	Peer leaders, local broadcasting station, social media, website
**Print media**	Posters, brochures	self-efficacy, attitude, knowledge	Churches, organizations, businesses	Peer Leaders, research team, social media, website
**Public Service Announcement**	Educational PSA of stroke preparedness	self-efficacy, attitude, social norms, knowledge	Flint community	radio
**Website and Facebook**	Dissemination of information, education, and materials	self-efficacy, attitude, social norms, knowledge	Flint community	Internet
**Mailers: magnet, brochure, information letter, action plan**	Dissemination of information and education, and materials	self-efficacy, attitude, social norms, knowledge	Flint community	United States Postal Service

Component 1: Peer-led workshop and workbook

Peer leaders will be hired from the community and trained to deliver the Stroke Ready workshop. The workshops, consisting of education related to stroke preparedness such as, a stroke narrative, stroke happens in Flint, good news: stroke is treatable, what is stroke, FAST signs, a tPA demonstration, timing is everything: call 911, Stroke Ready action plan, and Stroke Ready music video or audio, will last approximately 60 minutes.

Workshop materials that the peer-leaders will use to educate include a workbook, PowerPoint or flip chart/easel (pending availability of multi-media equipment per location), a role play script, a tPA demonstration, and an adapted music video for stroke education and preparedness. Interactive activities will include a role play, group discussions, and self-learning assessments. Each workshop will include education and behavioral/social learning strategies to which all educational materials have been geared. Participants may be given nominal gifts, such as a pens with the Stroke Ready logo.

For all materials and activities, if issues arise e.g., time constraints, the peer-leader may exclude some portions or alter formatting/order of certain sections in the workshop. These changes may be required in response to participant feedback or time constraints. Additionally, graphics may be slightly altered to enhance the professional, finished look of the product. None of the content of the materials should significantly change after IRB approval.

The peer-led workshop has been adapted so that it may be tailored in both length and interactive content, while maintaining integrity to the core public health message—stroke preparedness. These adaptations make the workshops more conducive to wide-spread implementation and allow for greater flexibility in provision of workshops given the needs and time-requirements of community organizations and community members. It is expected that about 150 workshops will be delivered throughout the 6-month time period per quadrant (x 4 quadrants).

While the full version of the workshop is the preferred version for delivery of the education, our community advisory board suggested a shorter option that may be more appropriate for the workplace or after established group gatherings. Thus, a 30-minute version of the workshop is an option. This abbreviated version of the workshop foregoes most of the interactive components such as the group discussion and role play.

Media format: (Pending availability of multi-media equipment per workshop location) Delivery of the intervention will be via a PowerPoint presentation with content from the workbook, audio recorded portions covering for core components, and music video.

Non-Media format: A flip chart/easel with content from the workbook will be used for delivery. Music from Stroke Ready video will be played on audio only, however peer leader will be able to facilitate participants viewing of the Stroke Ready music video on their personal smartphones, as well as directing participants to view it on the Stroke Ready website or Facebook page.

The workshop includes a workbook providing Flint-tailored stroke preparedness education and an audio recording to facilitate the content, as well as peer-led interactive activities. The core components of the Stroke Ready intervention include a stroke narrative, stroke happens in Flint, good news: stroke is treatable, what is stroke, FAST signs, a tPA demonstration, timing is everything: call 911, Stroke Ready action plan, and Stroke Ready music video or audio.

If a peer-led workshop is not feasible for a group, the study team may provide a shortened, non-peer-led version which includes core components of stroke preparedness education, brochures, and action plan, and the music video or instructions for accessing the music video. This “workshop” adaptation is about 10 -15 minutes long and it is expected to be a non-peer-led intervention to be disseminated throughout the 6-month time period per quadrant (x 4 quadrants).

Component 2: Stroke Ready Music Video

The Stroke Ready music video, an integral component of the Stroke Ready community intervention, was developed during the Stroke ready pilot (HUM00098718), incorporates the National Stroke Association’s FAST stroke symptoms pneumonic (e.g. F-facial droop, A-arm weakness, S—slurred speech, T—time to call 911) into an original gospel-based music score and video. There is also a strong focus on self-efficacy with the

video asking viewers to participate in demonstrating stroke signs.[29] There will be a full length and shorter version.

The Stroke Ready music video will be available to access on the Stroke Ready website and Facebook page and may be played during in the workshops pending media capabilities. Also, peer leaders will be asked to disseminate the Stroke Ready website address via their email, Facebook, and text messaging contacts. The link will also be placed on print materials.

Component 3: Print media – Posters and Brochures
The Stroke Ready program will include a print media campaign with posters and brochures to be dispersed by the research team per quadrant for display and distribution by local organizations. All print materials were developed with community input and feedback through focus groups and interviews with community members (HUM00130902). The posters focus on stroke symptoms and the importance of calling 911 among other stroke preparedness messages The brochure includes all core intervention components: stroke narrative, stroke happens in Flint, good news: stroke is treatable, what is stroke, FAST signs, tPA description, timing is everything: call 911, Stroke Ready action plan, and link to website where the Stroke Ready music video can be viewed.

Component 4: Broadcast Media – TV and Radio public service announcements (PSAs)
An about 60-second version of the music video and audio only version will be created for use as TV and radio PSAs. There will be two additional PSAs including stroke preparedness messages the research team developed using theory based health behavior change methods and reviewed by the community PI.

Component 5: Digital (Internet) Media – Website and Facebook
The Stroke Ready website will serve as a central repository for Stroke Ready information central to the Stoke Ready campaign, stroke information, and resources, Stroke Ready workshop information, printable versions of brochures and flyer-size versions of posters, links to community partner sites, and events in the Flint community. The Stroke Ready music video will be linked to the Stroke Ready website and Facebook page.

Component 6: Stroke Ready Mailers
The citywide intervention includes mailers printed in and distributed throughout the city of Flint on the per quadrant schedule. The mailer may include a Stroke Ready magnet, brochure, action plan, and information letter about the Stroke Ready campaign and where to find more information or a local stroke ready event.

Stroke Ready Fidelity Assessment, Process Evaluation, Outcomes
Fidelity assessment and process evaluation measures the extent to which an innovation and its activities are implemented as intended. As such, the research team will conduct fidelity assessments and collect process measures to track any changes or adaptations made to a Stroke Ready workshop and Stroke Ready campaign upon implementation. The Stroke Ready campaign will also collect primary and secondary outcomes (Table 4).

Fidelity Assessment
The research team will randomly select a proportion of workshops to observe for fidelity assessments to assess whether the workshop is being conducted as intended. Other fidelity measures the research team will collect include exposure of dose, quality of delivery, participant responsiveness, program differentiation, and intervention complexity. These assessments are to measure the degree to which teachers or staff are able to use the instructional practices as intended and facilitate the workshop as intended.

Aspects of fidelity the research team will measure (See Appendix A) include: dose delivered – as measured by workshop duration and content delivered; dose received – as measured by post-intervention survey to determine exposure to all intended activities per workshop type & satisfaction; quality of delivery – as measured by facilitator’s utilization of techniques prescribed by the program (use of facilitator’s guide, use of

audio/PowerPoint, appropriate facilitation of activities); reach – as measured by attendance; participant responsiveness – as measured by post-intervention survey to identify participant interest in activities and perceived usefulness of information; program differentiation – as measured through a post-intervention survey to identify which workshop components participants liked most; intervention complexity – as measured through tracking peer facilitator's attendance at initial training session and completion of refresher training if it has been longer than 6 months since the facilitator last delivered a workshop (which will be available as an online training module); and context – as measured through observation field notes of aspects of environment that may influence intervention implementation or study outcomes

Stroke Ready Process Evaluation
The research team will collect Stroke Ready community-level intervention and uptake process measures (Table 3). The community-level intervention uptake will be measured with questions in the community surveys, Speak to your Health and Flint Area Study, regarding exposure to and satisfaction with the posters, flyers, workbook and music video (See Appendix B). Uptake of the Stroke Ready video and internet materials will be measured by counts of internet hits from internet protocol (IP) addresses of video viewers from within Flint. This will be tabulated by linking to a database that maps IP address to physical locations. Satisfaction with the Stroke Ready workshop will be included in the participant survey (Appendix C).

Table 3: Process Measures

Community Intervention Process Measures	
**Uptake: Music video**	-Number of internet hits<br>-Number of internet hits from within Flint
**Uptake: Workshops**	-Number of workshop locations<br>-Number of workshops held (each type)<br>-Number of workshop participants
**Uptake: Print media**	-Number of mailers, action plans and brochures distributed
**Uptake: PSAs**	-Number of plays of PSAs on radio and TV
**Facebook uptake**	-Number of likes on Facebook page
**Website uptake**	-Number of hits to Stroke Ready website<br>- Number of hits to Stroke Ready website (from within flint)
**Uptake: Workbook**	-Number of materials distributed
**Community Satisfaction**	-Workshop survey<br>-FASt Survey<br>-Speak to Your Health Survey

Cost-effectiveness Analysis
The research team will assess the cost effectiveness of the overall Stroke Ready Program. Cost-effectiveness will be estimated for two intervention scenarios: Stroke Ready delivery and Stroke Ready development and delivery. This will inform the value of taking the Stroke Ready Intervention, "out of the box" and delivering it in a novel context and to separately assess the cost of developing and delivering a similar intervention in a novel context.

Cost inputs to the models will be carefully recorded throughout the project. For development costs, we will use budgets from the Stroke Ready pilot to estimate the cost of developing all materials used in this proposal — music video, workbooks, workshop content and print media. Delivery costs will be tracked with each phase of the Stroke Ready program. All Stroke Ready material expenditures (e.g. print media production, website maintenance) will be tracked, and as appropriate, assigned to either the hospital or community portion of the intervention. To track personnel time for the workshops, we will maintain a spreadsheet and update it after every Stroke Ready workshop with the number of individuals involved, including participants. Each hour spent on the grant will be mapped to costs by assigning appropriate job titles and then mapping hourly wages for that title to Bureau of Labor Statistics Survey wage survey data by profession.[30] By summing work time costs and material costs, we will be able to estimate the total costs of the overall intervention and the hospital and community interventions separately. We will then separately estimate total quality adjusted life years (QALYs) gained by the Stroke Ready program (and separately for the hospital and community interventions) by applying the primary outcome treatment effect size to the total hospitalized population (i.e. 2.2% increase in treatment rates * 500 strokes = 11 additional patients treated) and estimated QALY gain using published stroke cost effectiveness models.[31, 32] Estimated hospital and community effect sizes will be obtained from our secondary analysis assessing intervention component efficacy. The age distribution of patients who receive treatment via the intervention will be obtained from the overall Flint stroke population. By using repeated bootstrap samples from this population and repeatedly running the model we will estimate 95% credible intervals on the QALY

gain. We will then estimate the Incremental Cost Effectiveness Ratio (ICER) by dividing estimated costs / estimated QALY gain across all scenarios.

Outcome Measures

To both fully evaluate the Stroke Ready program and inform future interventions, we will measure the primary outcome and a number of secondary outcomes (Table 4); the process outcomes (intervention dissemination and reach) was described above. By measuring each step of the delivery process for the Stroke Ready campaign intervention we will collect secondary outcomes we hope will inform future CBPR stroke preparedness campaigns for community-level health education acute stroke treatment interventions.

Table 4. Stroke Ready Program Outcome Measures

Intervention Goal	Outcome Measure	Data Source	Data collection
Primary Outcome			
Increased acute stroke treatments	Acute stroke treatment rate	Flint Hospitals	2010-2021
Secondary Outcomes (Community Level)			
Optimize community for acute stroke	ED arrival by ambulance, onset to door time, door to needle time	Flint Hospitals	2010-2021
Improve self-efficacy, attitude, social norms	Self-efficacy, Attitude, subjective norm	Community Surveys	2015, 2017, 2019, 2021
Increase Stroke Preparedness	Stroke recognition, intent to call 911	Community Surveys	2015, 2017, 2019, 2021
Provide community stroke education	Exposure to local stroke education	Community Surveys	2019, 2021
Secondary Outcomes (Individual Level—Workshop Participants)			
Increase workshop Satisfaction	Satisfaction, Self-efficacy	Individual survey	During workshop

Ascertainment of Outcomes

The primary outcome will be measured from hospital EMR, billing data and/or from the Get With the Guidelines stroke registry data. Data will be separately received from the three hospitals in Flint (received letters of support and UFA in progress), which together account for 95% of all stroke treatments in Flint residents. The study population will be patients with a primary diagnosis of ischemic stroke using ICD-9 codes and/ or ICD-10 codes[33, 34] The primary outcome will be any thrombolysis which includes both IV tPA (MS-DRG 61-63 or ICD-9 procedure code 99.10), intra-artery treatment (MS-DRG 21-23 or CPT codes 37184-6, 37201, 75896) and the combination identified by ICD-9 and ICD-10 codes.[3] The research team will attempt to obtain data from 2010 to 2021 if available to account for linear trends.

The community secondary outcomes are the number of stroke patients who arrive by ambulance, onset to door time, and door to needle time. Data will be obtained from the EMR or GWTG data of the three hospitals in Flint. Additional community level secondary outcomes will be measured with community surveys. The community survey, Speak to Your Health, is a biennial, geo-coded survey that has been designed and administered by the Flint community, Genesee County Health Department since 2003.[35] We added questions from the Stroke Ready pilot (HUM00098718) (including stroke attitude, self-efficacy, social norms, and written stroke preparedness vignettes) to this community survey (see Appendix B) that was administered in 2015/2017 and will continue in 2019 and possibly 2021. The research team may add stroke education exposure questions to the 2019 and 2021 STYH surveys (see section 29 of IRB application- exposure questions). These additions, along with the original questions, will assess community level change in response to the Stroke Ready program; as well as exposure to local stroke education. Additionally, these same questions will be added to the Flint Area Study. This is a face to face interview of Flint residents. The survey data will be de-identified. STYH and FASt surveys are publically accessible.

Outcome analyses

<u>Primary Analysis: Determine impact of Stroke Ready Campaign on acute stroke treatment rates in Flint</u>

The primary analysis will be an interrupted time series comparison of acute stroke treatment rates in the three Flint hospitals. The pre-intervention period will be defined using EMR data prior to the start of the roll out of the community intervention. All patients admitted with a primary diagnosis of ischemic stroke in both the pre-intervention and intervention periods will be included in the primary analysis. Note: process measures evaluating the Stroke Ready campaign (quadrant crossover or music video view IP address) will not be linked to any obtained PHI. Logistic regression will be used to estimate the overall intervention efficacy (indicator variable) in a model predicting receipt of acute stroke treatment (binary variable). If a temporal trend exists in the pre-intervention period, we will adjust for the month since the start of the pre-period as a fixed effect while accounting for clustering at the hospital level. To maximize statistical power, both interventions (hospital-HUM00112536 and all community quadrants-this current protocol) will be parameterized with a single variable. With this approach, statistical power for the primary analysis will be more than adequate. Using hospital administrative and Medicare data, we estimate that at least 480 strokes per year will occur at the 3 Flint hospitals for a total of at least 1,440 strokes in the pre period and 1,800 in the post-period. Assuming a doubling in treatment rates (pre-intervention Medicare treatment rate 2.2%),[37] we will have over 90% power to detect this difference considering a two-sample binomial difference in proportions. This estimate is consistent with prior simulation work based on ARIMA analyses (effect size of 1.0 (pre-intervention monthly treatment rate = 2.2%, standard deviation = 2.1, predicted post-intervention treatment rate 4.3%, auto-correlation=0.3).[38]

<u>Secondary Analyses: Regional Comparisons and Quadrant-based Analyses to Enhance Causal Inference</u>

Secondary analyses will explore the extent that such confounding may influence the primary analysis and enhance the ability to draw causal inferences from the primary analysis. First, we will repeat the primary analysis with a concurrent control group consisting of other large Michigan metropolitan regions (regional control model) where African-Americans make up more than 25% of the population (Detroit, Saginaw, Muskegon, Benton Harbor). This analysis will control for regional effects that may lead to increased treatment rates that occur simultaneously with our intervention in Flint using data from the Michigan State Inpatient Database (SID),[39] which collects deidentified data on all acute care hospitalizations in the state of Michigan within a given year. Second, by delivering the intervention sequentially to geographic quadrants within Flint, we will explore whether increases in acute treatment rates parallel the geographic pattern of intervention roll out (geographic model). Specifically, each stroke patient in Flint will be geocoded to one of the four intervention quadrants using EMR data and a geocoding interface. Our primary analysis will then be repeated by modifying the intervention indicator variable to represent whether the intervention was active in the patient's geographic quadrant at the time of intervention.

<u>Exploratory Analyses: Efficacy of Program Components and Temporal Patterns to Inform Future Interventions</u>

To inform future interventions, the research team will perform a series of hypothesis-generating analyses to inform which elements of the program were most effective and the temporal properties of the program. Due to power concerns, our primary analysis does not consider the difference between the hospital and community effects. Thus, we will first estimate the proportion of the change in the acute stroke treatment rate attributable to the hospital-based intervention vs. the community-based intervention by repeating our geographic model including an indicator variable representing the time period of the hospital intervention as well as a community interaction term. In this way, we will be able to explore whether the Stroke Ready hospital or community based intervention was most efficacious and whether there was synergy between the interventions. Using simulation analyses, we estimate that there will be 70% power to find a doubling at the community level, 55% power to find a doubling at the hospital level and 21% power to find a doubling through a community-hospital interaction. Because this power is inadequate for a hypothesis-testing evaluation, we have specified this analysis as an exploratory analysis whose purpose is enhance our understanding of the importance of intervention elements and to inform future interventions. Second, we will determine the temporal properties of the Stroke Ready intervention by adding a linear slope term and exploring quadratic terms in our geographic model to estimate the time delay between intervention and changes in treatment rates and whether treatment rates level off or decline as the intervention persists into its latter years. Finally, a strength of our data collection approach is that we will be able to inexpensively assess the sustainability of intervention effect using Michigan SID data years

after the intervention is completed without needing to perform additional data collection. Together, such analyses will determine the sustainability of the intervention and inform future interventions.

Exploratory analyses: Secondary Outcomes and Exploratory Outcome analyses

Analyzing Secondary Outcomes and Process Measures

Changes in the proportion of patients arriving by ambulance over time will be assessed using logistic regression with an indicator variable representing the intervention period. Changes in the time from ED presentation to acute stroke treatment and door to treatment will be explored using linear regression with a similar indicator variable representing the intervention period. Second, changes in *secondary outcomes* measured with the community survey across intervention waves will be assessed with ordinal logistic regression (Likert-based outcomes) or chi-squared tests (binary outcomes) with indicator variables representing the survey wave. Process measures will be summarized with descriptive statistics, as pre-intervention values will be either unmeasurable or unintelligible, formal statistical comparisons will not be performed.

Human subjects

Protection of Human Subjects

Stroke Ready is a community-level stroke preparedness public health education campaign in Flint, Michigan. The PIs and other research team members take human subject protection extremely seriously.

We will use hospital data that could allow for identification of individuals (EMR address) thus we seek IRB approval as a "Standard, non-exempt, research project". Conversely, the majority of the remainder of the project should be considered a public health education and promotion campaign and thus fall out of regulatory oversight. Likewise, the research team is not collecting pre/post data on the educational activities or promotional materials adapted for the campaign; the original materials and content previously underwent a pre/posttest design (HUM00098718). The current materials and activities include the components in Table 2 and is the citywide public health stroke preparedness education portion of this study. The Stroke Ready workshop is free and open to public; and the workshop fidelity assessment is observation in nature. The research team will collect outcome and process measures (Tables 3 and 4) on the remainder of the campaign components: music video, PSAs, print materials, internet platform; the research team does not intend to link any of the secondary and process measures collected to the EMR data (primary outcomes).

Human Subjects Involvement, Characteristics and Design

The primary outcome of the research portion of the Stroke Ready program is to assess acute stroke treatment rates from the three Flint hospitals, Hurley Medical Center, McLaren-Flint and Genesys hospital, before and after a citywide, community-based public health education and promotion campaign. One way to do this is to decrease pre-hospital delay by increasing the proportion of stroke patients who arrive to the hospital via ambulance. Thus, public health education is provided to the Flint community on stroke warning signs and treatment availability via the Stroke Ready campaign. There are no exclusion criteria for the Stroke Ready materials or activities as community members can choose whether they wish to engage in the active (e.g. workshop) or passive (e.g. poster) components of Stroke Ready. The education is no more than minimal risk and the content is readily available stroke preparedness knowledge. All data, including primary and secondary outcomes, will be collected and protected by the research team. We will protect PHI with all our efforts. Since the hospital EMR data is PHI, this portion of the project will require a HIPAA authorization waiver.

Sources of Data

De-Identified

The community surveys, Speak to Your Health and the Flint Area Study, are publically available and deidentified. The Michigan State Inpatient Database (SID), which collects data on all acute care hospitalizations in the state of Michigan within a given year is also deidentified. Neither the workshop satisfaction survey nor the fidelity assessment will not include personal identifiers.

Identified

Hospital data will be obtained from the Flint area hospitals. Data will include basic demographics, treatments, stroke process measures, comorbidities, and outcomes of stroke patients. This data will include identifiable information in the form of addresses in order to map the stroke event from Flint into one of the four Stroke Ready quadrants. We expect some of this data to come from the EMR, billing records and Get With the Guidelines Stroke. We are working with each hospital to establish the optimal approach to obtaining the required data.

Additionally, a process measure is the number of views of the stroke ready music video. This will be assessed by the number of internet hits from within Flint via internet protocol (IP) addresses. The only use of this data is to assess the whether or not the video was viewed by and for the community it was untended.

Our community partners and peer-leaders will work with the research team to schedule workshop location and time convenient for the participants, peer leaders, and community partner(s). For purposes of tracking quadrant crossover, i.e. assess whether the workshop participant lives within the quadrant for which it was intended, the research team will collect participant address without any other identifiers, e.g. full name. The addresses will not be used to track or identify workshop participants, and the information will not be shared with members outside the research group. The address can be destroyed once the research team records within-quadrant workshop participation status. The research team or peer leaders may provide a courtesy reminder call several days before the workshop.

All paper data will be held strictly confidential in locked facilities and password protected computers/databases, only accessible by necessary members of the research team. All identifiable data will be stored on the secure and UMHS approved cloud storage, M-Box. The only researchers with access to the identifiable information in M-Box will be Drs. Skolarus, Burke and Feng. A deidentified dataset will be created from the identifiable data and the only researcher with access to this PHI link will be Drs. Skolarus, Burke and Feng. After developing de-identified datasets in the enclave environment, limited analytic datasets will be used for analysis. For all identifiable data collected, the research team will not use them to personally identify a Stroke Ready workshop participant, stroke patient, or Stroke Ready video viewer.

<u>Potential Risks</u>

The potential risk is possible disclosure of confidential personal health information. This risk is extremely low since the data will be stored on a secure non-networked computer and password protected. All data for analysis will be completely de-identified.

Physical risks: We do not anticipate our intervention will induce physical risks as it is all based on educational materials.

Psychological risks: We do not anticipate our intervention will induce psychological risks as we provide positive messages. We are not collecting sensitive health information that could lead to psychological harm if disclosed to unauthorized individuals.

Financial risks: We do not anticipate any financial burden all Stroke Ready education and activities are free to the public.

Legal risks: We do not anticipate that our research protocol will induce any additional legal risks.

<u>Stroke Ready Workshop and Materials</u>

The Stroke Ready workshop is community-based education with no more than minimal risk, participants will not be consented to observe or participant in the workshops.

Moreover, individuals who interact with the other educational components (print media campaign, mailing, video/ PSAs, workbook, website) will not be considered research participants and thus will not be consented as data will not be systematically collected for stroke preparedness effectiveness in a pre/posttest analysis. This was accomplished in the pilot (HUM0098718). They will receive the educational components of the intervention

but no data will be collected. The identity of the subject will not be able to be readily ascertained by the investigator or associated with the information.

Regarding identifiable data, we will request a HIPAA authorization waiver through the University of Michigan IRB and Flint hospital IRBs for the EMR. The research is not feasible without the waiver. The only patient identifier is address. In order to obtain HIPAA authorization from past and future patients, additional PHI information would be needed, such as patient name and address, which puts additional undo burden on the patient and greatly increases the risk of personal identification.

Protections Against Risk

All members of the research team have been trained in research ethics, confidentiality protection, and HIPAA prior to and throughout the study period through the Program for the Education and Evaluation in Responsible Research and Scholarship (PEERRS) training program at the University of Michigan Medical School. Any additional research personnel must also pass PEERRS certifications.

All paper data will be held strictly confidential in locked facilities and password protected computers/databases, only accessible by necessary members of the research team. All identifiable data will be stored on the secure and UMHS approved cloud storage, M-Box. The only researcher with access to the identifiable information in M-Box will be Drs.Skolarus, Burke and Feng. A deidentified dataset will be created from the identifiable data and the only researcher with access to this PHI link will be Drs. Skolarus, Burke and Feng. After developing de-identified datasets in the enclave environment, limited analytic datasets will be used for analysis. For all identifiable data collected, the research team will not use them to personally identify a Stroke Ready workshop participant, stroke patient, or Stroke Ready video viewer. Further, none of the secondary outcomes will be linked to the PHI in the primary outcome dataset.

The Stroke Ready workshop and materials do not have exclusion criteria, including age limits. This is a no more than minimal risk to the exposure to stroke education or participation in the workshop. We will work with the University of Michigan IRB to ensure that adequate provisions are made.

Potential Benefits of the Proposed Research to Human Subjects and Others

The goal of this project is to provide stroke preparedness education to increase acute stroke treatment. Participants will uniformly gain access to stroke knowledge and education. It is hoped that by increasing knowledge of stroke symptoms and stroke treatments, the community will be more likely to recognize a stroke and call 911 should they see it occur in their community. This will decrease time to hospital arrival and allow us to better care for future victims of stroke. The risks of this protocol are minimal in relation to the potential individual and societal benefits.

Importance of the Knowledge to be Gained

Primarily, this research study aims to increase acute stroke treatments in Flint, MI by providing health education and activities. It will allow us to design a citywide public health education stroke preparedness campaign. The risks of this protocol are minimal in relation to the potential individual and societal benefits of overall stroke education knowledge gain.

Inclusion of Women and Minorities

Given this is a community education intervention no one will be excluded from the community-level interventions. The Stroke Ready campaign should not be harmful to women or minorities.

Inclusion of Children

The Stroke Ready campaign should not be harmful to children as it is adapted from material delivered to youth in the pilot study (HUM00098718).

Vulnerable Populations

Given this is a community education intervention no one will be excluded from the community-level interventions. The Stroke Ready campaign should not be harmful to vulnerable population.

Data and Safety Monitoring Plan

Because there is a no more than minimal risk in the study, the PIs will provide data and safety oversight. Our local IRB will monitor the research portions of the project.

Sustainability and dissemination

Project sustainability will come through several mechanisms: 1) training of peer leaders who will have knowledge of stroke warning signs and the importance of calling 911; 2) a complete community intervention package that can be administered with little to no training; 3) a well-positioned CAB to promote sustainability; and 4) optimized acute stroke care in a safety net hospital. The products of this application include a strategy to improve acute stroke treatments in safety-net hospitals and an easy to deliver Stroke Ready community intervention to allow for successful of dissemination. If successful, this proposal will directly benefit the Flint community by increasing acute stroke treatment rates thereby decreasing post-stroke disability. Furthermore, it will inform future acute stroke interventions, especially in underserved, predominately African American communities.

References List

1. Streitfeld D. An effort to save flint, mich., by shrinking it. *New York Times*. 2009
2. **Impact of heart disease and stroke in michigan: 2008 report on surveillance. Lansing, mi: Michigan department of community health, bureau of epidemiology, chronic disease epidemiology section** [http://www.michigan.gov/documents/mdch/Impact_complete_report_245958_7.pdf] July 20, 2015
3. Skolarus LE, Meurer WJ, Shanmugasundaram K, Adelman EE, Scott PA, Burke JF. Marked regional variation in acute stroke treatment among medicare beneficiaries. *Stroke*. 2015;46:1890-1896
4. Barnes GD, Froehlich JB. Anticoagulation: Where we are and where we need to go. *J Thromb Thrombolysis*. 2008
5. Mozaffarian D, Benjamin EJ, Go AS, Arnett DK, Blaha MJ, Cushman M, et al. Heart disease and stroke statistics—2015 update a report from the american heart association. *Circulation*. 2015;131:e29-e322
6. **United states census bureau national population projections** [http://www.census.gov/population/projections/data/national/2012.html] September 10, 2013
7. Burke JF, Lisabeth LD, Brown DL, Reeves MJ, Morgenstern LB. Determining stroke's rank as a cause of death using multicause mortality data. *Stroke*. 2012;43:2207-2211
8. Pandya A, Gaziano TA, Weinstein MC, Cutler D. More americans living longer with cardiovascular disease will increase costs while lowering quality of life. *Health affairs*. 2013;32:1706-1714
9. Wilkinson PR, Wolfe CD, Warburton FG, Rudd AG, Howard RS, Ross-Russell RW, et al. A long-term follow-up of stroke patients. *Stroke*. 1997;28:507-512
10. Kelly-Hayes M, Beiser A, Kase CS, Scaramucci A, D'Agostino RB, Wolf PA. The influence of gender and age on disability following ischemic stroke: The framingham study. *Journal of stroke and cerebrovascular diseases : the official journal of National Stroke Association*. 2003;12:119-126
11. Tissue plasminogen activator for acute ischemic stroke. The national institute of neurological disorders and stroke rt-pa stroke study group. *N Engl J Med*. 1995;333:1581-1587
12. Berkhemer OA, Fransen PS, Beumer D, van den Berg LA, Lingsma HF, Yoo AJ, et al. A randomized trial of intraarterial treatment for acute ischemic stroke. *N Engl J Med*. 2015;372:11-20
13. Goyal M, Demchuk AM, Menon BK, Eesa M, Rempel JL, Thornton J, et al. Randomized assessment of rapid endovascular treatment of ischemic stroke. *N Engl J Med*. 2015;372:1019-1030
14. Campbell BC, Mitchell PJ, Kleinig TJ, Dewey HM, Churilov L, Yassi N, et al. Endovascular therapy for ischemic stroke with perfusion-imaging selection. *N Engl J Med*. 2015;372:1009-1018
15. McDermott M, Skolarus LE, Burke JF. A systematic review of interventions to increase tpa administration. *Stroke*. 2016;47:A181-A181

16. Soulleihet V, Nicoli F, Trouve J, Girard N, Jacquin L. Optimized acute stroke pathway using medical advanced regulation for stroke and repeated public awareness campaigns. *Am J Emerg Med*. 2014;32:225-232
17. Fonarow GC, Zhao X, Smith EE, Saver JL, Reeves MJ, Bhatt DL, et al. Door-to-needle times for tissue plasminogen activator administration and clinical outcomes in acute ischemic stroke before and after a quality improvement initiative. *JAMA*. 2014;311:1632-1640
18. Van Schaik SM, Van der Veen B, Van den Berg-Vos RM, Weinstein HC, Bosboom WM. Achieving a door-to-needle time of 25 minutes in thrombolysis for acute ischemic stroke: A quality improvement project. *Journal of stroke and cerebrovascular diseases : the official journal of National Stroke Association*. 2014;23:2900-2906
19. Quain DA, Parsons MW, Loudfoot AR, Spratt NJ, Evans MK, Russell ML, et al. Improving access to acute stroke therapies: A controlled trial of organised pre-hospital and emergency care. *The Medical journal of Australia*. 2008;189:429-433
20. O'Brien W, Crimmins D, Donaldson W, Risti R, Clarke TA, Whyte S, et al. Faster (face, arm, speech, time, emergency response): Experience of central coast stroke services implementation of a pre-hospital notification system for expedient management of acute stroke. *Journal of clinical neuroscience : official journal of the Neurosurgical Society of Australasia*. 2012;19:241-245
21. Moynihan B, Davis D, Pereira A, Cloud G, Markus HS. Delivering regional thrombolysis via a hub-and-spoke model. *Journal of the Royal Society of Medicine*. 2010;103:363-369
22. De Luca A, Toni D, Lauria L, Sacchetti ML, Giorgi Rossi P, Ferri M, et al. An emergency clinical pathway for stroke patients--results of a cluster randomised trial (isrctn41456865). *BMC health services research*. 2009;9:14
23. Morgenstern LB, Bartholomew LK, Grotta JC, Staub L, King M, Chan W. Sustained benefit of a community and professional intervention to increase acute stroke therapy. *Archives of internal medicine*. 2003;163:2198-2202
24. Wojner-Alexandrov AW, Alexandrov AV, Rodriguez D, Persse D, Grotta JC. Houston paramedic and emergency stroke treatment and outcomes study (hopsto). *Stroke*. 2005;36:1512-1518
25. Mellon L, Doyle F, Rohde D, Williams D, Hickey A. Stroke warning campaigns: Delivering better patient outcomes? A systematic review. *Patient Relat Outcome Meas*. 2015;6:61-73
26. Boden-Albala B. Results of citywide intervention among african-americans to improve stroke preparedness and ed arrival time. *142nd APHA Annual Meeting and Exposition (November 15-November 19, 2014)*. 2014
27. Gardois P, Booth A, Goyder E, Ryan T. Health promotion interventions for increasing stroke awareness in ethnic minorities: A systematic review of the literature. *BMC Public Health*. 2014;14:409
28. Hemming K, Haines T, Chilton P, Girling A, Lilford R. The stepped wedge cluster randomised trial: Rationale, design, analysis, and reporting. *BMJ*. 2015;350:h391
29. Skolarus LE, Zimmerman MA, Murphy J, Brown DL, Kerber KA, Bailey S, et al. Community-based participatory research: A new approach to engaging community members to rapidly call 911 for stroke. *Stroke*. 2011;42:1862-1866
30. Vazquez Ruiz de Castroviejo E, Marquez Garcia A, Fajardo Pineda A, Lozano Cabezas C, Guzman Herrera M, Ramirez Moreno A, et al. [patterns of clinical presentation of atrial fibrillation in hospitalized patients]. *Rev Esp Cardiol*. 2003;56:1187-1194
31. Fletcher JJ, Kotagal V, Mammoser A, Peterson M, Morgenstern LB, Burke JF. Cost-effectiveness of transfers to centers with neurological intensive care units after intracerebral hemorrhage. *Stroke*. 2015;46:58-64
32. Leppert MH, Campbell JD, Simpson JR, Burke JF. Cost-effectiveness of intra-arterial treatment as an adjunct to intravenous tissue-type plasminogen activator for acute ischemic stroke. *Stroke*. 2015;46:1870-1876
33. Tirschwell DL, Longstreth WT, Jr. Validating administrative data in stroke research. *Stroke*. 2002;33:2465-2470
34. Goldstein LB. Accuracy of icd-9-cm coding for the identification of patients with acute ischemic stroke: Effect of modifier codes. *Stroke*. 1998;29:1602-1604
35. Shirey LA, Griffith DM, Brady J, Kruger DJ, Morrel-Samuels S, Greene-Moton E. Challenges and lessons learned in developing a community-based health survey. *Progress in community health partnerships : research, education, and action*. 2008;2:99-104
36. Wyse DG. Anticoagulation in atrial fibrillation: A contemporary viewpoint. *Heart Rhythm*. 2007;4:S34-39
37. Skolarus LE, Meurer WJ, Shanmugasundaram K, Adelman EE, Scott PA, Burke JF. Marked regional variation in acute stroke treatment among medicare beneficiaries. *Stroke*. 2015;46:1890-1896

38. Zhang F, Wagner AK, Ross-Degnan D. Simulation-based power calculation for designing interrupted time series analyses of health policy interventions. *Journal of clinical epidemiology*. 2011;64:1252-1261
39. Rubboli A, Di Pasquale G. The combination of anticoagulant and anti-platelet therapy in patients with atrial fibrillation: A comment on the recent acc/aha/esc guidelines for the management of patients with atrial fibrillation. *Eur Heart J*. 2006;27:2908-2909; author reply 2909-2910

Appendix

Appendix A:

Stroke Ready Fidelity Observation Form

Peer leader name: ____ Date/time: _____/_____:___

Workshop Location: ____1st evaluation: o Yes o No

Number of participants ________________

**Logistics**	**Yes**	**No**	**Explain**
Did the workshop start on time?			
Did the workshop end on time?			
Did all participants stay for the entire workshop?			
Did all attendees sign in?			
Did each participant receive a workbook and pen?			
Did each participant receive a certificate of completion?			

Please rate the following workshop activities.

**Implementation**	**None**	**Some (less than ½)**	**Most (more than ½)**	**All**	**Significant aspects missed (If "some" or "most" was checked)**
How many F.A.S.T. stroke signs did the peer trainer(s) review?					
How many participants engaged in the F.A.S.T. stroke					

signs review?					
How many participants engaged in the role play activity?					
How many reasons for not calling 911 did the peer leaders discuss?					
How many participants engaged in the review questions at the end of the workshop?					

	**Yes**	**No**	**Explain**
Did all participants sign the action plan?			
If participants were reluctant to participate in activities, did the leaders gently encourage, but NOT force their participation (even if unsuccessful)?			

Please answer the following about the peer leader(s).

**Facilitator/Participation**	**Strongly Agree**	**Agree**	**Neither Agree/Disagree**	**Disagree**	**Strongly Disagree**
Facilitator had a professional appearance					
Facilitator’s guide was used for delivery					
Did the leader leave out or skip over content material provided in the leaders’ manual?					
Did the facilitator appear knowledgeable?					
Did the facilitator appear welcoming?					
The facilitator appear to make the					

workshop more interesting?					
Was the leader able to keep participants on topic?					
Participants respond well to facilitator					
Read text from the training manual word-for-word					
Asked questions to the participants as instructed in the training manual					
Encouraged participant discussion after asking questions					
Played audio clips in their entirety from the PowerPoint					
Played video clips in their entirety from the PowerPoint					
When using personal examples, did the leaders talk about themselves for less than one minute?					

**<u>Setting</u>**	**Strongly Agree**	**Agree**	**Neither Agree/Disagree**	**Disagree**	**Strongly Disagree**
The setting was conducive to workshop delivery (i.e. participants could see/hear facilitator, easel and audio/visual clips)					

General notes: Please note if any slides were skipped during the workshop. Please note the length of time for the workshop, whether the audio/video clips were loud and easy to hear throughout the room, and if there were any technical difficulties with the PowerPoint.

Appendix B:

These questions will appear in the Speak to your Health and Flint Area Survey community-level surveys.

<u>Please circle the ONE option below for each question that BEST describes how you feel (only circle ONE)</u>

**1. Medical treatment can help someone having a stroke.**				
1	2	3	4	5
Strongly agree	*Agree*	*neither agree/nor disagree*	*Disagree*	*Strongly disagree*

**2. If I saw someone having a stroke, I would know what to do.**				
1	2	3	4	5
Strongly agree	*Agree*	*neither agree/nor disagree*	*Disagree*	*Strongly disagree*

**3. Most people would call 911 if they saw someone having a stroke.**				
1	2	3	4	5
Strongly agree	*Agree*	*neither agree/nor disagree*	*Disagree*	*Strongly disagree*

4. Which of the following are signs of a stroke? (Check the box for all that apply)

θ Sudden chest pains
θ Sudden arm weakness
θ Sudden nose bleed
θ Sudden stomach pains
θ Sudden face drooping
θ Sudden trouble talking
θ Sudden coughing hard

What is the first thing you would you do if you saw…

5. **While walking to the store, your sister suddenly dropped her purse and could not pick it back up. (Please check one box)**

    - θ Wait a couple of hours, then decide
    - θ Call a family member or friend immediately
    - θ Call Doctor's office immediately
    - θ Call 911 immediately

6. **While out to lunch, your friend is suddenly confused and unable to order from the menu. (Please check one box)**

    - θ Wait a couple of hours, then decide
    - θ Call a family member or friend immediately
    - θ Call Doctor's office immediately
    - θ Call 911 immediately

7. **After helping to move furniture, your friend tells you his arms hurt and feel weak. (Please check one box)**

    - θ Wait a couple of hours, then decide
    - θ Call a family member or friend immediately
    - θ Call Doctor's office immediately
    - θ Call 911 immediately

Appendix C:

Hello Stroke Ready Participant,

Thank you for taking part in the Stroke Ready workshop! This survey is a chance for you to tell us what you thought about the Stroke Ready workshop.

If you have any questions about the survey, please ask the project staff.

Thank you for helping improve the Stroke Ready program!

<u>Section A</u>

The questions in this section are to get your feedback on what you thought about the Stroke Ready workshop.

**Please rate how much you agree with the following statements**					
Please circle one answer per statement					
	**Strongly**	**Agree**	**Neither agree, nor**	**Disagree**	**Strongly**

	agree		disagree		disagree
If I saw someone having a stroke, I would know what to do.	5	4	3	2	1
Everyone in Flint should know this information.	5	4	3	2	1
I am satisfied with the workshop.	5	4	3	2	1

**Please rate how much did you liked each of the following activities**						
Please circle one answer per activity						
**Activity**	**This was one of my least favorite activities.**	**One of the more boring activities, but not one of my least favorites**	**It was okay**	**One of the better activities, but not one of my favorite activities.**	**This was one of my favorite activities**	**This activity was not included in my workshop**
	**The Worst!**	→			**The Best!**	**N/A**
a. Signs of Stroke Role Play	1	2	3	4	5	0
b. Discussion of barriers to calling 911	1	2	3	4	5	0
c. TPA demonstration	1	2	3	4	5	0
d. Stroke Ready music video/song	1	2	3	4	5	0

786

**If we offered the Stroke Ready workshop again, would you tell a friend they should attend?**		
o No	o Maybe	o Yes

Why? ____

____

Now we have a few questions about you----

Section B

Please mark the answer that best describes you.

Are you...

θ Man

θ Woman

How old are you? _________

What is your race?

θ Black or African American

θ White or European American

θ Other *(please write in)*: _______________________

Are you Hispanic *(optional)*?

θ Yes

θ No

θ I'm not sure

How much school have you completed? (please mark all that apply)

θ Less than High School

θ High School graduate/GED

θ Trade School

θ Some college

θ College or University graduate

θ Advanced degree

Have you or someone you know had a stroke? Check all that apply.

θ Yes, I have had a stroke.

θ Yes, someone I know has had a stroke.
θ No

Thanks so much for participating in Stroke Ready and completing this survey!!
