# Supplementary material for: Association of the Stroke Ready Community-Based Participatory Research Intervention With Incidence of Acute Stroke Thrombolysis in Flint, Michigan
Source: JAMA Netw Open. 2023 Jul 3;6(7):e2321558. doi: 10.1001/jamanetworkopen.2023.21558 (PMC10318478; doi:10.1001/jamanetworkopen.2023.21558)
Supplement: Supplement 3. — Data Sharing Statement [file jamanetwopen-e2321558-s003.pdf]

Data Sharing Statement

Skolarus. Association of the Stroke Ready Community-Based Participatory Research Intervention With Incidence of Acute Stroke Thrombolysis in Flint, Michigan. *JAMA Netw Open*. Published July 03, 2023. doi:10.1001/jamanetworkopen.2023.21558

Data
Data available: No
